# MitoCOGs: clusters of orthologous genes from mitochondria and implications for the evolution of eukaryotes

**DOI:** 10.1186/s12862-014-0237-5

**Published:** 2014-11-25

**Authors:** Sivakumar Kannan, Igor B Rogozin, Eugene V Koonin

**Affiliations:** National Center for Biotechnology Information, National Library of Medicine, National Institutes of Health, Bethesda, MD 20894 USA

**Keywords:** Mitochondria, Genome evolution, Gene loss, Gene transfer, Introns, Clusters of orthologous genes

## Abstract

**Background:**

Mitochondria are ubiquitous membranous organelles of eukaryotic cells that evolved from an alpha-proteobacterial endosymbiont and possess a small genome that encompasses from 3 to 106 genes. Accumulation of thousands of mitochondrial genomes from diverse groups of eukaryotes provides an opportunity for a comprehensive reconstruction of the evolution of the mitochondrial gene repertoire.

**Results:**

Clusters of orthologous mitochondrial protein-coding genes (MitoCOGs) were constructed from all available mitochondrial genomes and complemented with nuclear orthologs of mitochondrial genes. With minimal exceptions, the mitochondrial gene complements of eukaryotes are subsets of the superset of 66 genes found in jakobids. Reconstruction of the evolution of mitochondrial genomes indicates that the mitochondrial gene set of the last common ancestor of the extant eukaryotes was slightly larger than that of jakobids. This superset of mitochondrial genes likely represents an intermediate stage following the loss and transfer to the nucleus of most of the endosymbiont genes early in eukaryote evolution. Subsequent evolution in different lineages involved largely parallel transfer of ancestral endosymbiont genes to the nuclear genome. The intron density in nuclear orthologs of mitochondrial genes typically is nearly the same as in the rest of the genes in the respective genomes. However, in land plants, the intron density in nuclear orthologs of mitochondrial genes is almost 1.5-fold lower than the genomic mean, suggestive of ongoing transfer of functional genes from mitochondria to the nucleus.

**Conclusions:**

The MitoCOGs are expected to become an important resource for the study of mitochondrial evolution. The nearly complete superset of mitochondrial genes in jakobids likely represents an intermediate stage in the evolution of eukaryotes after the initial, extensive loss and transfer of the endosymbiont genes. In addition, the bacterial multi-subunit RNA polymerase that is encoded in the jakobid mitochondrial genomes was replaced by a single-subunit phage-type RNA polymerase in the rest of the eukaryotes. These results are best compatible with the rooting of the eukaryotic tree between jakobids and the rest of the eukaryotes. The land plants are the only eukaryotic branch in which the gene transfer from the mitochondrial to the nuclear genome appears to be an active, ongoing process.

**Electronic supplementary material:**

The online version of this article (doi:10.1186/s12862-014-0237-5) contains supplementary material, which is available to authorized users.

## Background

The mitochondrion is a membrane-bounded organelle that performs multiple, pivotal roles in the eukaryotic cell. The primary function of the mitochondria is the synthesis of ATP through the oxidative electron transport chain but mitochondria are also involved in other biological functions such as intracellular signaling including induction of programmed cell death [1-3]. Although for many years several groups of protists have been considered primary amitochondriate forms, the current consensus is that all extant eukaryotes possess either typical mitochondria or organelles that appear to be derived mitochondria such as mitosomes or hydrogenosomes [4-6]. Mitochondria (but not mitosomes or most hydrogenosomes) possess their own genome, albeit a drastically reduced one, and a translation machinery that translates the mRNAs transcribed from the mitochondrial genes to synthesize a small but essential subset of mitochondrial proteins [7,8].

It is considered firmly established that the mitochondria in all eukaryotes are monophyletic and that the ancestor of all extant mitochondria originated from a unique endosymbiotic event that occurred over a billion years ago [9,10]. Phylogenetic analyses unequivocally indicate that the endosymbiont that gave rise to the mitochondria was an alpha-proteobacterium, most likely affiliated with rickettsia and/or the SAR11 group [9,11-14]. A recent genomic survey focused on the evolution of bioenergetic pathways has suggested that the closest extant relatives of the mitochondria are methylotrophic alpha-proteobacteria such as *Rhodobacterales* [15]. The mitochondrial endosymbiosis undoubtedly was a pivotal event in eukaryogenesis and later in the origin of multicellular life forms. Two classes of hypotheses have been proposed with regard to the host of the endosymbiont and the subsequent evolutionary scenario [6]. The “archezoan” hypotheses postulate that the host was a proto-eukaryote with already developed eukaryotic features, such as the advanced endomembrane system, including the nucleus, the cytoskeleton and the phagocytic capacity that enabled the engulfment of the alpha-proteobacterial endosymbiont [16-19]. The alternative, symbiogenetic hypotheses posit that the host was a prokaryote, most likely an archaeon, and the endosymbiotic event triggered the emergence of the hallmark eukaryotic features of cellular organization including the nucleus [6,20-24]. Regardless of the exact nature of the host or the endosymbiont, it is clear that this unique event has dramatically and permanently altered the course of eukaryotic evolution.

After endosymbiosis, mitochondria followed the path of reductive evolution both in terms of the organelle structure and functions, and the genome. In several lineages, the mitochondria have severely degenerated to become hydrogenosomes or mitosomes [5,25,26]. These derived mitochondria-like organelles have lost the ability to generate ATP by oxidative phosphorylation with oxygen as the terminal acceptor although hydrogenosomes can generate ATP by substrate phosphorylation [26]. The only known function of mitosomes is their involvement in iron-sulfur cluster assembly [27].

Given the bacterial ancestry of mitochondria, one might expect the mitochondrial proteome to be of bacterial origin. However, the mitochondrial proteome is a complex mosaic of proteins of diverse origins [28,29]. Many proteins that function in the mitochondria indeed appear to originate from bacteria although mostly not from known alpha-proteobacteria [30,31]. In addition, several key proteins involved in replication and transcription of the mitochondrial genome are of bacteriophage origin [32,33] whereas a large number of proteins do not have identifiable orthologs outside the eukaryotic lineage [34-36]. The non-alpha-proteobacterial origins of a large fraction of mitochondrial proteins might have to do with the fluidity of bacterial genomes because of which the gene complement of the mitochondrial ancestor could have been substantially different from those of any of the extant alpha-proteobacteria [31]. Recently, the “pre-endosymbiont” hypothesis has been proposed under which the mitochondrial proteins of non-alpha-proteobacterial origin were already present and functional in an endogenously formed organelle in the eukaryotic host cell and were adopted by the proto-mitochondria following endosymbiosis [37].

After endosymbiosis, most of the genes of the endosymbiont were lost or transferred from the endosymbiont to the nuclear genome. Even the most gene-rich, bacteria-like mitochondrial genomes of certain jakobids, such as *Andalucia* and *Reclinomonas*, encompass a maximum of 106 genes of which 72 are protein-coding [38,39] compared to at least several hundred genes in the smallest alpha-proteobacterial genomes, even those of endosymbionts [40].

It has been estimated that at least 1,500 proteins contribute to the maintenance and functioning of mammalian mitochondria [41]. The mitochondrial genome encodes only a miniscule fraction of these proteins (as few as 3 in the apicomplexan *Plasmodium falciparum* and as many as 72 in the jakobid *Andalucia goyodi*) whereas the nuclear genome encodes the rest of the proteins that are synthesized in the cytosol and imported into the mitochondria. Given this dominance of nuclear-encoded proteins, it is not immediately clear why mitochondria retain their genome. Several explanatory hypotheses have been proposed. (1) Proteins that are encoded in the mitochondrial genome are extremely hydrophobic which would hamper their import into mitochondria. This reasoning might account for some but not for all proteins that are encoded in the mitochondrial genome [42]. (2) Some mitochondria, for example those of metazoa, employ a non-standard genetic code for translation [43]. These genes, if transferred to the nuclear genome, cannot be translated by the cytosolic ribosomes that use the standard genetic code. Again, however, this does not hold true for mitochondrial genomes of many other eukaryotes, e.g. plants, that use the standard genetic code. (3) Potentially the most convincing hypothesis, known as colocation for redox regulation (CoRR), states that the protein-coding genes that remain in the mitochondrial genome are required to be located close to the site of oxidative phosphorylation for regulation of their expression depending on the redox state of electron carriers in the electron transport chain [44,45].

As pointed out above, the gene content in mitochondrial genomes varies from 3 to 106 genes (not counting various mobile elements). Accumulation of sequenced mitochondrial genomes from thousands of eukaryotic species creates unprecedented opportunities for a comprehensive reconstruction of the mitochondrial genome evolution across the history of the eukaryotic domain. Such reconstruction can help addressing several fundamental problems that remain unsolved such as the extent of parallel gene loss and gene gain in mitochondrial evolution, evolution of gene structure after the transfer of mitochondrial genes to the nucleus, and more. Clusters of Orthologous Genes (COGs) have proved to be a powerful framework for functional annotation of new genomes as well as comparative genomic and evolutionary studies [46-49]. Here we describe Clusters of Orthologous Genes for Mitochondrial-encoded Proteins (MitoCOGs) using all available mitochondrial-encoded proteomes. We employ the MitoCOGs to analyze the phyletic distribution of mitochondrial-encoded proteins and specifically to identify proteins that are not encoded in the largest known jakobid mitochondrial genomes but are encoded in other mitochondrial genomes. MitoCOGs are also used to identify the nuclear-encoded mitochondrial genes that were transferred from the mitochondrial genome to the nuclear genome in some species and to explore the evolution of the structure of these genes.

## Methods

### Protein sequences encoded in mitochondrial genomes

36,120 protein sequences from 2,486 complete mitochondrial genomes with representatives from all the major eukaryotic supergroups were used to generate the MitoCOGs. Incomplete kinetoplastid proteomes were obtained from GOBASE [50] and all other protein sequences were obtained from the NCBI non-redundant protein sequence database [http://www.ncbi.nlm.nih.gov/genomes/GenomesHome.cgi?taxid=2759&hopt=html]. The analyzed complete genomes grouped by taxonomy are listed in Additional file 1: Table S1.

### Construction of MitoCOGs

MitoCOGs were generated using a slightly modified procedure for COG construction that was described previously [48,49]. Briefly, all-against-all BLASTP searches were conducted for the 35,593 protein sequences with a requirement that the reciprocal best hits cover at least 50% of both the query and the subject protein sequences. Initial clusters of symmetrical best hits were constructed using the COG construction software [51]. This initial clustering yielded 175 clusters that included 33,684 of the 35,593 sequences of mitochondria-encoded proteins.

### Expanding the initial clusters

Due to the strict requirement of the 50% coverage threshold and only a single best hit in each species, the COG construction software missed shorter sequences and paralogs. The initial clusters were expanded to include these sequences as follows. A position-specific scoring matrix (PSSM) for each initial cluster was created by aligning cluster member protein sequences using MUSCLE [52], followed by using the PSSMs as query for PSI-BLAST searches against the database of 35,593 mitochondrion-encoded protein sequences with an e-value threshold of 0.01. The protein sequences with similarity above the cut-off that were not previously included in the initial clusters were added to their corresponding best-scoring initial clusters. Subsequently, when new mitochondrial genomes became available, they were added to the MitoCOGs in a similar manner (335 sequences from 11 genomes). In addition, for the sake of completeness, proteins that are encoded in only two or even a single species were also included in the MitoCOGs (6 sequences from 5 genomes). Altogether 1,056 sequences were added to the initial clusters.

### Merging the expanded clusters

The strict requirements for COG creation also result in underclustering when a COG is split into two or more clusters. To remedy over-splitting, the expanded clusters were merged using a modification of the procedure described previously [48]. The PSSMs for the expanded clusters were generated by aligning the member sequences using MUSCLE followed by using the PSSMs as queries for PSI-BLAST searches against the database of all mitochondrial sequences in the expanded clusters with an e-value threshold of 0.01. The PSI-BLAST hits were grouped by the cluster they belong to and self-hits were excluded. Using the PSI-BLAST score for each hit, a mean score was calculated for each cluster and only the best-scoring cluster was considered. If two clusters showed best scores to one another, these clusters were then merged after examination. This procedure was performed iteratively until the clusters cannot be merged further. This merging procedure resulted in 119 MitoCOGs from the original 175 sequence clusters. Additional 21 MitoCOGs were created manually bringing the total number of MitoCOGs to 140 and the sequences to 34,751.

### Reconstruction of ancestral gene content of mitochondrial genomes

Ancestral gene content evolution of mitochondrial genomes was reconstructed using the Count software [53]. For a given species tree and a phyletic distribution of genes for these species, Count infers ancestral gene content by posterior probabilities in a phylogenetic birth-and-death model. The program computes the probability for a gene to be present at ancestral nodes and the sum of these probabilities gives the estimate of the ancestral gene content. Three putative eukaryotic species trees of 43 species were used, with the root placed (1) between unikonts and bikonts [54,55], (2) between excavates and the rest of eukaryotes [56], (3) between jakobids and the rest of the eukaryotes [56]. The phyletic distribution of 71 MitoCOGs was represented as an absence/presence matrix (encoded as 0/1). Because gene gain in mitochondrial genome is rare, a pure-loss model architecture was assumed and the prior distribution at the root was assumed to be Poisson. For a given species tree, Count also estimates the number of lineages that have lost a particular gene. By grouping the genes based on their biological function (for example, all genes encoding proteins that are part of Complex 1 of the electron transport chain), the average propensity for the complex to be lost during the evolution was calculated.

### Identification of nuclear-encoded orthologs of MitoCOGs

Identifying the nuclear-encoded orthologs of MitoCOGs is a non-trivial task because nuclear genomes encompass genes coding for homologs of mitochondrial proteins (e.g. cytosolic and plastid ribosomal proteins) that can be difficult to distinguish from bona fide mitochondrial proteins. In order to identify the true mitochondrial orthologs, MitoCOGs PSSMs were searched using PSI-BLAST (with an e-value threshold of 1e-4 and a coverage threshold of 30%) against complete proteomes of selected eukaryotes (nuclear-encoded), alpha-proteobacteria, cyanobacteria and archaea (see Additional file 1: Tables S4-7). If the number of mitochondrion-encoded sequences was too small to generate a PSSM, a protein BLAST (BLASTP with an e-value threshold of 1e-4 and a coverage threshold of 30%) search was performed. The PSI-BLAST/BLASTP hits along with the corresponding MitoCOG sequences were aligned using MUSCLE, followed by removing the poorly aligned columns with either GBlocks or trimAL [57,58]. Then, a maximum likelihood tree was generated for each alignment using PhyML. Finally, the trees were manually inspected and only the sequences that formed a clade with mitochondrial-encoded and alpha-proteobacterial sequences were selected as likely nuclear-encoded orthologs of MitoCOGs.

### Subcellular localization prediction

Subcellular localization for the nuclear-encoded mitochondrial genes was predicted using TargetP v1.1 [59] and MitoProt II v1.101 [60]. TargetP predicts the likely subcellular location of a protein based on the presence of N-terminal target peptides. MitoProt predicts the probability of a protein being imported into mitochondria using N-terminal target sequence and hydrophobicity characteristics of the protein.

### Analysis of intron locations

Intron-exon boundaries for nuclear-encoded mitochondrial genes were determined using WebScipio [61]. Scipio takes a protein sequence as input and searches against the corresponding genome sequence using BLAT [62]. Intron density was calculated as the number of introns per 1 kb of coding sequence.

To estimate the number of shared and species- or lineage-specific introns among the orthologous genes, orthologous amino acid sequences were aligned using MUSCLE and this alignment was used as a guide to align their corresponding nucleotide sequences using TranslatorX [63]. Intron boundaries were mapped onto this alignment and shared positions were defined as introns occurring at exactly the same nucleotide in the multiple alignment essentially as described previously.

### Reconstruction of intron gain and loss events

For the reconstruction of intron gain and loss scenarios in the nuclear-encoded genes, intron positions were represented as a data matrix of intron absence/presence (encoded as 0/1). The matrices of intron absence/presence along with the corresponding species tree were used as the input data for the DOLLOP program of the PHYLIP package [64]. This program employs the Dollo parsimony approach, which is based on the assumption that each derived character state (in this case, intron presence) originated only once on the tree [65]. The states of intron presence–absence in internal nodes, including the root of the eukaryotic tree as well as the number of intron gains and losses for each branch, were derived from the DOLLOP output using an ad hoc program. The alignments, matrices of intron presence–absence and phylogenetic trees for nuclear-encoded genes analyzed in this work are available at ftp://ftp.ncbi.nih.gov/pub/koonin/MitoCOGs.

### Phylogenetic analyses

Phylogenetic analyses were performed using (1) only mitochondrion-encoded proteins and (2) both mitochondrion-encoded and nuclear-encoded proteins. Alpha-proteobacterial sequences were used as outgroup. Protein sequences of individual MitoCOGs (and nuclear-encoded proteins for the second dataset) were aligned with the alpha-proteobacterial sequences using MUSCLE and the poorly aligned columns were removed as previously described [66]. The alignments were then concatenated. Maximum-likelihood analyses were performed using PhyML [67]. PROTTEST [68] was used to select the best-fitting substitution model for the concatenated supermatrix according to Akaike information criteria. PROTTEST estimated that substitution model “LG” with “+G” (estimated distribution of the gamma shape parameter) and “+F” (estimated amino acid frequencies by counting the occurrence of the different amino-acids in the alignment) as the best model for these datasets.

## Results

### MitoCOGs

Clusters of orthologous protein-coding genes located in mitochondrial genomes and their orthologs relocated to nuclear genomes (MitoCOGs) were generated as described under Methods. Altogether, 140 MitoCOGs were delineated from 34,751 mitochondrial-encoded proteins. Additional file 1: Figure S1 shows the maximum, minimum, and median coverage for mitochondrial genomes grouped by taxonomy. Most of the mitochondrial genomes of animals, ascomycete fungi, and apicomplexa were covered almost completely, with only a few exceptions. For most taxa, the median coverage was above 90% except for the most gene-rich excavate mitochondrial genomes (*Malawimonas, Naegleria, Andalucia* and *Reclinomonas*). However, there are at least 8 mitochondrial genomes with coverage below 50% (Additional file 1: Table S2). Mitochondrial genes of slime mold *Physarum polycephalum* undergo extensive RNA editing and thus the protein sequences that are directly translated from the DNA sequence are poorly covered by MitoCOGs [69]. The low coverage in *Moniliophthora perniciosa* and land plants is due to the presence of multiple species-specific hypothetical proteins.

Additional file 1: Table S3 shows the functional breakdown of the MitoCOGs. Out of the 140 MitoCOGs, 73 are typical mitochondrial proteins, 17 are proteins that are usually encoded in introns or mobile elements, and the rest 49 are proteins with unknown functions. For the sake of completeness, proteins that are encoded in only two or even a single species were also included in the MitoCOGs. All MitoCOGs with unknown functions are lineage-specific, with ciliates having the largest number of uncharacterized MitoCOGs (19) followed by streptophytes (18) (Additional file 1: Tables S9 and S10).

### Phyletic distribution of MitoCOGs

**Figure 1:**
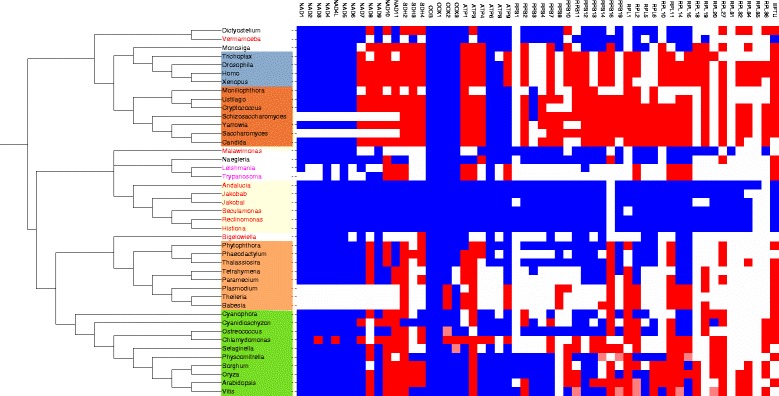
**Phyletic distribution of MitoCOGs (blue) and their nuclear-encoded orthologs (red).** Light red color indicates that the protein is encoded in both mitochondrial and nuclear genomes. White color indicates an orthologous protein is either absent or cannot be identified. For species with names shown in red, only mitochondrial proteomes are used; the species with names shown in pink, the mitochondrial proteomes are incomplete. Protein names and abbreviations: NADH dehydrogenase subunits 1–11, 4L (NAD[1-11], 4L), succinate dehydrogenase subunits 2–4 (SDH[2-4]), cytochrome-c oxidase subunits 1–3 (COX[1-3]), cytochrome b (COB), ATP synthase subunits 1, 3, 4, 6, 8, 9 (ATP[1, 3, 4, 6, 8-9]), ribosomal protein small subunits 1–4, 7, 8, 10–14, 16, 19, VAR1 (RPS[1-4, 7, 8, 10-14, 16, 19], VAR1), ribosomal protein large subunits 1, 2, 5, 6, 10, 11, 14, 16, 18, 19, 20, 27, 31, 32, 34, 35, 36 (RPL[1, 2, 5, 6, 10, 11, 14, 16, 18-20, 27, 31, 32, 34, 35, 36]), elongation factor EF-Tu (EF-Tu).

The phyletic distribution of 56 MitoCOGs involved in oxidative phosphorylation and protein translation across the eukaryotic taxa is shown in blue in Figure 1. With the notable exception of several genes involved in oxidative phosphorylation, mitochondrial gene content varies significantly, especially for the ribosomal proteins. The mitochondrial gene content varies even within some taxa. For example, the gene content among the mitochondria from the taxonomic groups Glaucophyta and Chlorophyta varies significantly. Genes that are usually encoded in mitochondria (NAD2, NAD4L, NAD7, COX2, COX3) are transferred to the nuclear genome in the green alga *C. reinhardtii* (Figure 1). In contrast, animal and fungal mitochondrial genomes encode almost the same set of genes except for the RPS3 and VAR1 genes in some fungi and the TatC gene in some animals.

### The jakobid mitochondrial genomes as a superset of eukaryotic mitochondrial genes

**Figure 2:**
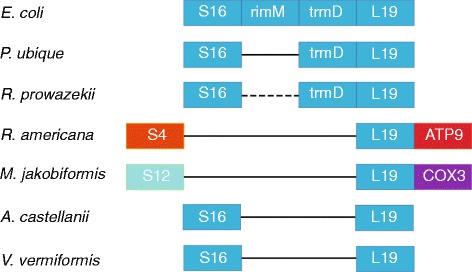
**Conservation of ribosomal proteins (RPS16 and RPL19) gene order among amoebas** ***V. vermiformis*** **and** ***A. castellanii*** **, excavates** ***R. americana*** **and** ***M. jakobiformis*** **,** ***E. coli*** **, and alphaproteobacteria** ***P. ubique*** **and** ***R. prowazekii*** **.** Notably, RPS16 gene is not encoded in the mitochondrial genome of *R. americana*.

The most gene-rich *A. goyodi* mitochondrial genome encodes 66 functionally characterized proteins and 6 species-specific predicted proteins with unknown functions. The mitochondrial proteins that are unique to jakobids are bacterial type RNA polymerase (RNAP) subunits alpha (rpoA), beta (rpoB), beta-prime (rpoC), and sigma (rpoD), ribosomal large subunit proteins RPL1, RPL27 and RPL34, RPL35 and COX15 (the latter two present only in *A. goyodi*), SecY protein involved in co-translational membrane translocation of proteins (absent only in *A. goyodi*), and the 6 uncharacterized proteins. The RNAP function in other eukaryotes is relegated to a single subunit bacteriophage-type polymerase that is encoded in the nuclear genome [32,70]. Conversely, at least 3 proteins (excluding the proteins that are encoded in mobile elements) are missing in the jakobids with the most gene-rich mitochondrial genomes but are encoded in the mitochondrial genomes of one or more of the other eukaryotes. Specifically, the DNA mismatch repair protein MutS is encoded in sponge mitochondrial genomes, DNA adenine methylase (DAM) in the haptophyte *Emiliania huxleyi*, and the ribosomal small subunit protein RPS16 in amoeba *Vermamoeba* (formerly *Hartmannella*) *vermiformis* and the excavate *Malawimonas jakobiformis*. The MutS [71] and DAM [72] genes might have been acquired by mitochondrial genomes via horizontal gene transfer but in the case of RPS16 this route of evolution appears unlikely. In addition to RPS16, *V. vermiformis* also encodes the ribosomal large subunit protein RPL19 and elongation factor EF-Tu that are otherwise present only in jakobids. Notably, the genes for RPS16 and RPL19 in *V. vermiformis* show similar order to that in the *trmD* operon of several alpha-proteobacteria and other bacteria (Figure 2). Recently, highly diverged genes encoding RPS16 and RPL19 proteins have been identified in the mitochondrial genome of the related species, *Acanthamoeba castellanii* [73].

### Reconstruction of ancestral gene content of mitochondrial genomes

**Figure 3:**
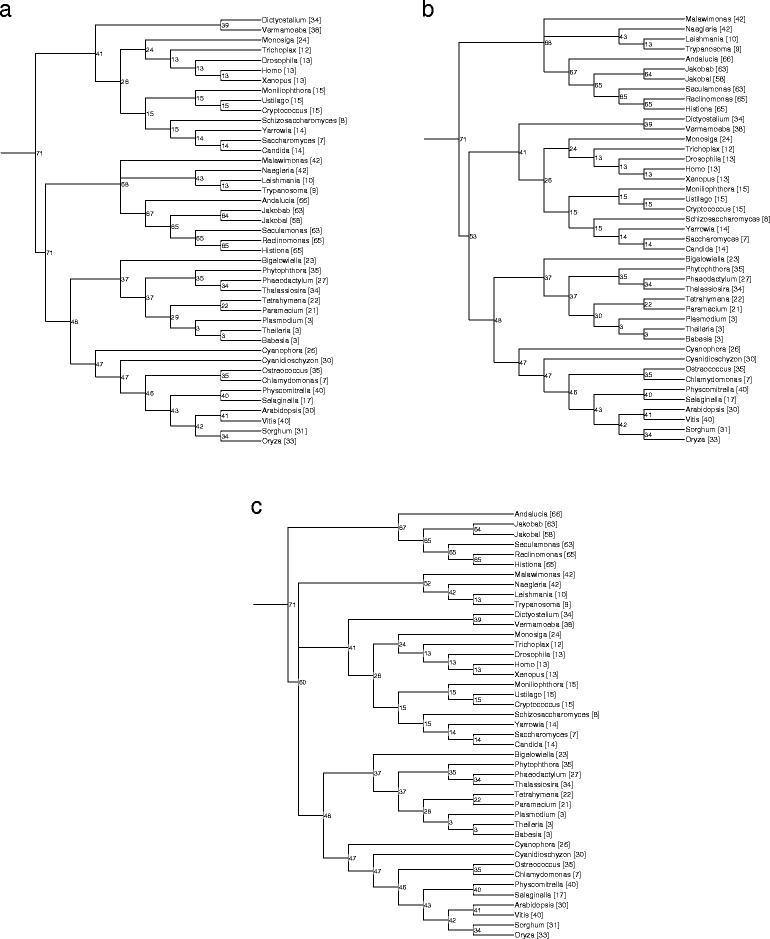
**Reconstruction of ancestral mitochondrial gene sets using MitoCOGs. (a)** A species tree with the eukaryotic root between the unikonts and bikonts was employed as the framework for the reconstruction. **(b)** A species tree with the eukaryotic root between excavates and the rest of the eukaryotes was employed as the framework for the reconstruction. **(c)** A species tree with the eukaryotic root between the jakobids and the rest of the eukaryotes was employed as the framework for the reconstruction. The numbers in parentheses after the taxon name is the number of MitoCOGs genes encoded in the mitochondrial genome. Numbers in each internal node indicate the inferred size of ancestral gene content.

We used three alternative eukaryotic species trees, with the root positioned either (1) between the unikonts and bikonts [54,55], (2) between excavates and the rest of eukaryotes [56], (3) between jakobids and the rest of the eukaryotes [56], and the phyletic distribution of the mitochondrial-encoded genes to obtain maximum likelihood reconstructions of the ancestral gene content using Count (see Methods). This analysis mapped 71 genes to the last common ancestor of extant mitochondrial genomes (Figure 3). The scenario with the jakobids as the basal branch minimized the number of parallel gene losses compared to the other two scenarios, with the minimal losses occurring in the jakobids but 11 genes lost on the stem of the second eukaryotic subtree (compare Figure 3c with Figure 3ab)

From the Count analysis, we estimated the average propensity of loss (transfer) for individual mitochondrial proteins and multisubunit complexes (by averaging the number of lineages that have lost the genes that constitute the complex). The lineages that have lost individual genes and complexes are listed in Additional file 1: Table S8. All analyzed genes were estimated to have been independently lost more than twice except for COB and COX1. The most frequently lost gene is the one encoding ribosomal protein S10 that appears to have been independently lost in 13 or 14 lineages (depending on the topology of the eukaryotic tree). Overall, small subunit ribosomal proteins show the highest propensity to be lost followed by the succinate dehydrogenase complex (complex II). The mean propensity of gene loss for large subunit ribosomal proteins is much lower compared to small subunit ribosomal proteins (Additional file 1: Table S8). Cytochrome b and the cytochrome c oxidase subunits have the lowest propensity to be lost from the mitochondrial genomes.

### Nuclear orthologs of MitoCOGs

Lineage-specific transfer of mitochondrial genes to the nuclear genome results in patchy phyletic patterns of MitoCOGs when only genes present in mitochondrial genomes are included (Figure 1). Putative nuclear-encoded orthologs of MitoCOGs were identified by searching the database of nuclear-encoded proteins with MitoCOG PSSMs using PSI-BLAST followed by phylogenetic analyses. For this analysis, we only considered the 56 MitoCOGs that include proteins involved in oxidative phosphorylation and protein translation. A total of 970 nuclear-encoded homologs in 55 species were identified for these MitoCOGs (Additional file 1: Figures S2 and S3).

The phyletic distributions of MitoCOGs and their nuclear-encoded orthologs are shown jointly in Figure 1. Of the 56 analyzed mitochondrial genes, 45 show a “dual” distribution, i.e. each of these genes resides in the mitochondrial genome in some species but in the nuclear genome in other species. The sea urchin *Strongylocentrotus purpuratus* had the maximum number of nuclear orthologs (33) that mapped to 21 MitoCOGs. However, most of the redundant hits could be due to errors in gene prediction [74]. Otherwise, fungal species possess the maximum number of identifiable orthologs for MitoCOGs, especially for the ribosomal proteins. The ribosomal proteins in metazoa are probably too diverged to be identified by using mitochondrion-encoded proteins as queries [75]. Indeed, mammalian nuclear-encoded mitochondrial ribosomal proteins have been estimated to evolve 13 times faster than the cytoplasmic ribosomal proteins in the same cell [76]. However, using sensitive profile Hidden Markov Models searches and annotations from the literature, additional 347 nuclear-encoded mitochondrial proteins (mostly ribosomal) were added to the MitoCOGs. In the plantae supergroup, the green alga *Chlamydomonas reinhardtii* has the largest number of nuclear-encoded orthologs (24) followed by *Arabidopsis thaliana* (23). *C. reinhardtii* is exceptional in that some of the genes that encode proteins involved in oxidative phosphorylation that are typically encoded in the mitochondrial genome have been transferred to the nuclear genome. In contrast, another green alga, *Ostreococcus tauri,* encodes most of its genes in the mitochondrial genome, with only 7 identifiable genes encoded in the nuclear genome. *Naegleria gruberi* whose mitochondrial gene content is the closest to the most gene-rich jakobid mitochondrial genomes in our dataset had only 3 identifiable nuclear-encoded orthologs. The analyzed dataset included 5 nuclear genomes from species that lack regular mitochondria: *Encephalitozoon cuniculi, Entamoeba histolytica, Giardia lamblia, Nosema ceranae,* and *Trichomonas vaginalis*. None of these species encompass any identifiable orthologs of mitochondrial genes.

Among the MitoCOGs, ATP3 shows the widest phyletic distribution of nuclear orthologs (47 of the 55 species) followed by SDH2 (46 of the 55 species) (Additional file 1: Figure S3). For several MitoCOGs, no nuclear-encoded orthologs were identified. This lack of nuclear orthologs could be due to two reasons: (1) these proteins are predominantly encoded by mitochondrial genomes such as most of the genes involved in oxidative phosophorylation, (2) these proteins are too diverged to be confidently identified by using MitoCOGs as queries (e.g. ribosomal proteins). Nuclear-encoded mitochondrial ribosomal protein content dramatically varies among the eukaryotes (mammalian mitoribosomes share only 74% of the yeast and 43% of the Kinetoplastid mitoribosomal proteins). Many mammalian ribosomal proteins have diverged significantly and have increased in size compared to their alphaproteobacterial ancestors and thus making it difficult to identify them with MitoCOG profiles [75].

For 14 genes, both nuclear and mitochondrial versions were identified in the same genome (Figure 1). Of these, 12 duplicated genes are found in land plants, one gene in the green alga *Ostreococcus tauri* and one in the fungus *Giberella zeae* (Figure 1). The enrichment of duplicated mitochondrial genes in land plants is statistically significant (P = 9.2 × 10^−7^, 2×2 Fisher exact test, 12 vs. 2 genes were compared with 6 species of land plants vs. 40 other species excluding Apicomplexa and amitochondrial eukaryotes). However, only one such duplication, that of the RPL2 gene, is conserved in two species (*Vitis vinifera* and *Arabidopsis thaliana*). Such high variability of the double nuclear-mitochondrial encoding is consistent with the hypothesis that functional gene transfer from the mitochondrial genome to the nuclear genome is an ongoing process in land plants [42,77,78]. Indeed, mitochondria in many land plants are engaged in transfer of DNA between and within species. Recently, it has been shown that, although the magnitude of horizontal gene transfer (HGT) involving nuclear genes is appreciable in parasitic plants, HGT involving mitochondrial genes is much more frequent [79]. This finding is consistent with several previous studies which suggest that plant genomes have undergone frequent HGT events, especially in the mitochondrial genome [80-86]. Parasitic plants provide the strongest evidence of HGT that appears to be facilitated by the intimate physical association between the parasites and their hosts [79,87-89]. The HGT appears to occur only in individual taxa and involves only some of the mitochondrial genes, suggesting that the fixation of these transfers occurs at the single gene level [79,84-89]. Some of the species-specific duplicated genes detected here are likely to represent recent transfers known as NUclear-encoded MiTochondrial-origin sequences (NumtS) [90]. For example, for 6 of the 7 *A. thaliana* genes that are duplicated in the mitochondrial and nuclear genomes, the encoded protein sequences are more than 99% identical, and so are the sequences of the only pair of duplicated genes in *O. tauri*. However, several of these nuclear genes, the high sequence similarity with the mitochondrial counterparts notwithstanding, contain introns that obviously have been inserted after the transfer (see also below).

### Phylogenetic analysis of mitochondrial genes

Nuclear-encoded mitochondrial proteins along with MitoCOGs have been proposed as alternative phylogenetic markers for resolving the tree of eukaryotes [91]. We constructed phylogenetic trees from concatenated alignments of two datasets; the mitochondrial-encoded proteins only and mitochondrial-encoded proteins complemented with nuclear-encoded mitochondrial proteins, with alpha-proteobacteria as an outgroup. Both approaches recovered the monophyly of major eukaryotic groups (Additional file 1: Figures S5 and S6). However, phylogenetic analysis failed to show consistent support for the grouping of Malawimonas with Opisthokonta which has been suggested previously based on the phylogenies of some mitochondrial proteins [91]. In contrast, we obtained a strong support for the basal position of jakobids [56] in the case when only mitochondrial-encoded proteins were analyzed (Additional file 1: Figure S5).

However, phylogenetic trees of mitochondrial proteins showed numerous deviations from well-established features of the eukaryotic phylogeny, e.g. grouping of the green alga Chlamydomonas with chromalveolates and plants with Excavates (Additional file 1: Figures S5 and S6). Furthermore, phylogenetic positions of some species (e.g. Dictyostelium) showed a substantial deviation from the expected placement within unikonts, suggesting that various phylogenetic artifacts create major problems for accurate tree reconstruction from mitochondrial protein sequence alignments [92-96]. Most likely, these difficulties are caused primarily by the erratic change of evolutionary rates in mitochondrial genomes from different eukaryotic lineages.

### Comparison of exon-intron structures of nuclear-encoded orthologs of mitochondrial genes

Exon-intron structure for the nuclear-encoded orthologs of MitoCOGs was determined using WebScipio. WebScipio produced predictions of the intron-exon structures for 783 of the 970 sequences whereas 38 sequences gave no BLAT match when searched against their corresponding genome sequences and 149 sequences returned incomplete results and were excluded from further analyses.

Many nuclear orthologs of mitochondrial genes are intronless, followed by genes with only one intron (Additional file 1: Figure S7). In contrast, the NAD7 gene in sea urchin *Strongylocentrotus purpuratus* has 17 introns although, as mentioned above the prediction of intron-exon boundaries for *Strongylocentrotus purpuratus* should be taken with caution [74].

**Figure 4:**
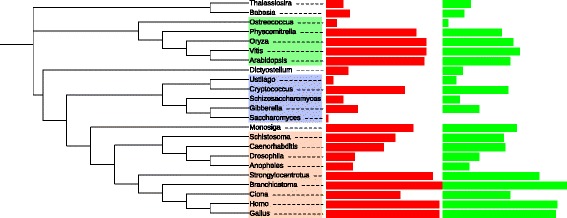
**Comparison of intron densities of nuclear-encoded mitochondrial genes and ancestral eukaryotic genes.** Green, mitochondrial genes; red, ancestral nuclear genes. The bar lengths correspond to the intron density. The p-value is based on a chi-squared test comparing the frequency of introns in nuclear-encoded mitochondrial genes and ancestral eukaryotic genes to the expected frequency calculated using the number of positions in nuclear-encoded mitochondrial genes and ancestral eukaryotic genes. The p-values for individual species are as follows: *Anopheles gambiae* (0.74), *Arabidopsis thaliana* (0.01), *Babesia bovis* (0.91), *Branchiostoma floridae* (0.76), *Caenorhabditis elegans* (0.82), *Ciona intestinalis* (0.69), *Cryptococcus neoformans* (0.27), *Dictyostelium discoideum* (0.94), *Drosophila melanogaster* (0.67), *Gallus gallus* (0.91), *Gibberella zeae* (0.41), *Homo sapiens* (0.90), *Monosiga brevicollis* (0.38), *Oryza sativa* (0.05), *Ostreococcus taurii* (0.53), *Physcomitrella patens* (0.00), *Saccharomyces cerevisiae* (0.22), *Schistosoma mansoni* (0.55), *Schizosaccharomyces pombe* (0.90), *Strongylocentrotus purpuratus* (0.54), *Thalassiosira pseudonana* (0.31), *Ustilago maydis* (0.12), *Vitis vinifera* (0.09).

We compared the intron densities (the number of introns per 1 kb of coding sequence) in the nuclear orthologs of mitochondrial genes with the mean intron densities of the respective nuclear genomes which were taken from the previous analysis [97] (Figure 4). The intron densities of the nuclear-encoded predicted mitochondrial sequences and the mean intron densities of their corresponding genomes were very similar for all studied species except for the land plants in which the intron densities of the mitochondrial genes were significantly lower than the mean intron density (Figure 4). A previous study has shown that the intron densities of chloroplast-derived genes were slightly, but significantly lower than those in other genes of land plants [98]. However, for the mitochondria-derived genes analyzed here, the difference in intron density compared to nuclear genes was much greater, about 1.5-fold (Figure 4). Intron densities in nuclear-encoded mitochondrial genes can be used as a proxy to date the transfer event from the mitochondrial genome to the nuclear genome [99]. The significantly lower intron density in all studied land plants (including moss, Figure 4) suggests that a substantial fraction of these genes were transferred more recently compared to chloroplast-derived genes. In general, our results are consistent with the hypothesis that functional gene transfer from the mitochondrial genome is an ongoing process in land plants [42,77,78].

The majority of the introns (47.5%) were of phase 0, followed by phase 1 (29.7%) and phase 2 (22.7%) (Additional file 1: Figure S8). The non-uniform distribution of intron phases with abundance of phase 0 introns and rarity of phase 2 introns with phase 1 being intermediate with an approximate ratio of 5:3:2 in mitochondrial genes is similar to the intron phases in nuclear genomes [100,101].

Depending on the phases of the flanking introns, exons can be partitioned into symmetric (0–0, 1–1, 2–2) and asymmetric (0–1, 0–2, 1,0, 1–2, 2–0, 2–1). Symmetric 0–0 exons are most common in eukaryotic genomes followed by 1–0 exons [101]. Although 0–0 exons accounted for majority of the mitochondrial genes (26%), the second most common was 2–0 (15%) rather than 1–0 (8.5%) (Additional file 1: Figure S9). This is in contrast to the estimation for mitochondrial genes in another study [99] though the gene set used in that study is slightly different.

**Figure 5:**
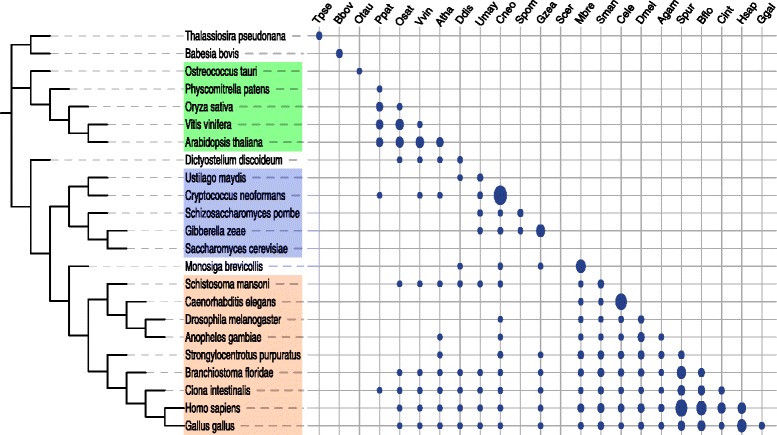
**Lineage-specific and shared introns among selected eukaryotes from diverse lineages.** In the matrix diagram, the rows are linked to the phylogenetic tree of eukaryotes and show the number of intron positions that are species-specific (diagonal elements, i.e. the same species in row and column) and those that are shared between species. The species names in the columns are abbreviated versions of the species names in the rows. See the legend to Figure 6 for species names and their abbreviations. The size of each ellipse reflects the number of intron positions.

Introns are mostly conserved among closely related species with fewer species-specific introns and even fewer shared introns between distant lineages (Figure 5). The species with most species-specific introns are *Chlamydomonas reinhardtii* (73%), *Cryptococcus neoformans* (72%) and *Caenorhabditis elegans* (72%), which belong to plantae, fungi, and metazoa groups, respectively. Among distant lineages of eukaryotes, there are few shared introns in nuclear orthologs of mitochondrial genes. For example, only 3 of the 105 introns in these genes are shared between human and Arabidopsis, in a sharp contrast to approximately 30% conserved introns in ancestral eukaryotic genes.

### Reconstruction of intron gain and loss events

We applied Dollo parsimony to reconstruct the most parsimonious scenario for the evolution of gene structure [65,102], i.e. the distribution of intron-gain and intron-loss events across the tree branches, in the nuclear orthologs of the mitochondrial genes. The use of Dollo parsimony as opposed to more complex maximum likelihood models [97,103-105] seems to be justified, in this case, because maximum likelihood analysis would not be reliable due to the small number of potential gains and losses. We cannot rule out the contribution of parallel gain of introns in the orthologous sites in independent lineages (this is one of the assumptions of Dollo parsimony, see Methods for details), however, the impact of parallel gain is expected to be small [106,107].

**Figure 6:**
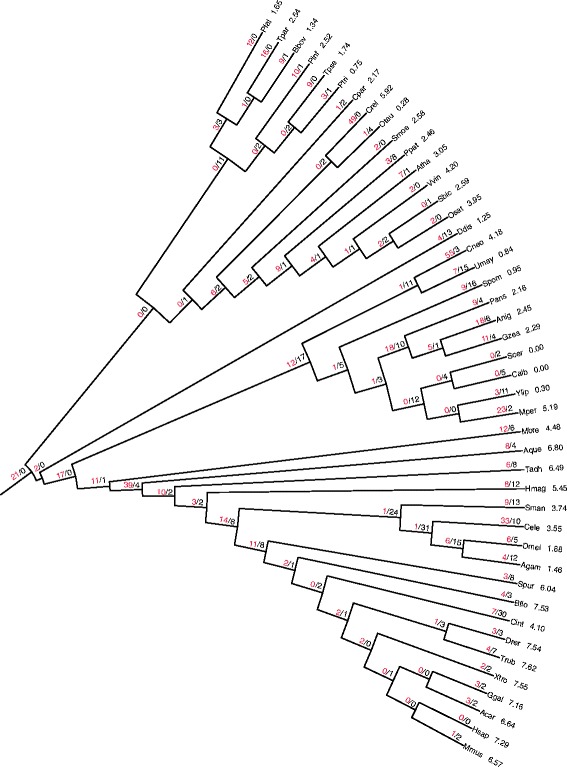
**Reconstruction of intron gain and losses among the nuclear genes encoding mitochondrial proteins.** Intron density of mitochondrial genes for each species is given after the species name. Species names and abbreviations: *Amphimedon queenslandica* (Aque), *Anolis carolinensis* (Acar), *Anopheles gambiae* (Agam), *Arabidopsis thaliana* (Atha), *Aspergillus niger* (Anig), *Babesia bovis* (Bbov), *Branchiostoma floridae* (Bflo), *Caenorhabditis elegans* (Cele), *Candida albicans* (Calb), *Chlamydomonas reinhardtii* (Crei), *Ciona intestinalis* (Cint), *Cryptococcus neoformans* (Cneo), *Cyanophora paradoxa* (Cpar), *Danio rerio* (Drer), *Dictyostelium discoideum* (Ddis), *Drosophila melanogaster* (Dmel), *Gallus gallus* (Ggal), *Gibberella zeae* (Gzea), *Homo sapiens* (Hsap), *Hydra magnipapillata* (Hmag), *Moniliophthora perniciosa* (Mper), *Monosiga brevicollis* (Mbre), *Mus musculus* (Mmus), *Oryza sativa* (Osat), *Ostreococcus tauri* (Otau), *Phaeodactylum tricornutum* (Ptri), *Physcomitrella patens* (Ppat), *Phytophthora infestans* (Pinf), *Plasmodium falciparum* (Pfal), *Podospora anserina* (Pans), *Saccharomyces cerevisiae* (Scer), *Schistosoma mansoni* (Sman), *Schizosaccharomyces pombe* (Spom), *Selaginella moellendorffii* (Smoe), *Sorghum bicolor* (Sbic), *Strongylocentrotus purpuratus* (Spur), *Takifugu rubripes* (Trub), *Thalassiosira pseudonana* (Tpse), *Theileria parva* (Tpar), *Trichoplax adhaerens* (Tadh), *Ustilago maydis* (Umay), *Vitis vinifera* (Vvin), *Xenopus tropicalis* (Xtro), *Yarrowia lipolytica* (Ylip).

Under the resulting parsimonious scenario, only 21 (3%) introns were already present in the nuclear orthologs of the mitochondrial genes of the last common ancestor of the analyzed eukaryotic species (Figure 6). This low fraction of conserved intron positions contrasts the previously obtained estimates for ancestral eukaryotic genes, where the fraction of ancestral introns has been estimated to exceed 13% [97,102-104]. These findings are consistent with the hypothesis that at least in some lineages the gene flow from the mitochondria to the nucleus continued after the divergence of the major groups of eukaryotes. Except for this remarkable observation, the overall picture of intron gain-losses is similar to that for the highly conserved nuclear genes analyzed in previous studies [97]. There was a substantial intron gain at the branches leading to the ancestor of metazoans but no comparable intron gain or loss in animals except in Ciona (Figure 6). There was a relatively slow accumulation of introns in the land plants whereas some branches of fungi apparently experienced extensive intron gain and loss (Figure 6).

## Discussion

The MitoCOGs described here are a resource for analysis of the genes that are present in at least one sequenced mitochondrial genome but in many eukaryotes have been transferred to the nuclear genome. Altogether there are 70 such genes, not counting genes of numerous mobile elements that are integrated in mitochondrial genomes of many plants, fungi and diverse unicellular eukaryotes. These genes represent a relatively small subset of the endosymbiont-derived component of the mitochondrial proteome as most of the retained endosymbiont genes have been transferred to the nucleus at an early stage of the evolution of eukaryotes and have not been identified in the mitochondrial genome of any extant eukaryotes [29,35,36,108,109]. The mitochondria-encoded subset is clearly non-random in terms of gene functions and appears to represent a distinct stage of mitochondrial evolution. Functionally, this group of genes consists primarily of two categories: i) genes for components of electron transfer complexes and ii) genes for components of the translation system. All the universal genes in mitochondrial genomes belong in the first category, conceivably because of the requirements of coupling between production and redox regulation of the respective proteins as stipulated by the CoRR hypothesis [1,44,45]. The encoding of protein components of the translation system in mitochondrial genome is not essential given that in numerous eukaryotes these genes have been relocated to the nuclear genome. Nevertheless, it appears that a gene complement that included many genes for translation system components along with the genes for RNAP subunits was an early intermediate stage in the evolution of mitochondria. A recent comparative analysis of the genome reduction trajectories in mitochondria and chloroplasts has revealed extensive convergence in the loss versus retention of ribosomal protein genes [110]. The retained ribosomal proteins are primarily involved in the ribosome subunit assembly. Accordingly, it has been speculated that the retention of these key r-proteins in the organelle genome remains advantageous for efficient ribosome assembly in situ but this requirement is gradually lifted with the shrinking of the rRNA during evolution such that all r-protein genes are transferred or lost in animals that experience the ultimate reduction of rRNA [110]. The massive parallel loss of r-protein genes correlated with the shrinking of rRNA reported here is compatible with this hypothesis. A comparison of rRNA size and the number of ribosomal protein coding genes in mitochondrial genomes showed that the suggested threshold of 3.4 kb rRNA size below which the organelle genomes loses all ribosomal protein coding genes [110] holds true for metazoan and some alveolate mitochondrial genomes (Additional file 1: Figure S10).

The existence of a relatively gene-rich intermediate in mitochondrial evolution is implied by the fact that nearly all mitochondrial gene sets are different subsets of the mitochondrial gene complement of the jakobids. Comparative analysis of the mitochondrial gene sets so far yielded a single clear exception to this pattern, the presence of the RPS16 gene in the mitochondrial genome of the amoebas *Vermamoeba vermiformis* and *Acanthamoeba castellanii* that is missing in the excavates. The history of RPS16 conceivably is the most striking case of massive parallel gene loss in mitochondrial evolution. Given the lack of evidence of reintroduction of genes into mitochondrial genomes and the alpha-proteobacteria-like arrangement of the r-protein genes, including RPS16, in *V. vermiformis* and *A. castellanii* (Figure 2), it appears virtually certain that the RPS16 gene comes from the ancestral endosymbiont. Accordingly, the history of this gene included multiple parallel losses, in particular one in the stem of the excavate branch.

Although most mitochondrial genomes contain many fewer genes than that of jakobids*,* only 10 proteins (including 4 RNAP subunits) are unique to the jakobids. Thus, it appears most likely that the mitochondrial gene set of jakobids is close to the ancestral state of the eukaryotic mitochondrial genome. Clearly, this ancestral state is far removed from the full genome of the alpha-proteobacterial ancestor of the mitochondria: judging by the smallest genomes of the extant alpha-proteobacteria that might be affiliated with the proto-mitochondrial lineage, such as *Rickettsia* and *Pelagibacter ubique*, the genome of the original endosymbiont encompassed about 1000 genes if not more. The great majority of these genes were either lost or transferred to the nuclear genome, conceivably in a rapid succession, at early stages of the evolution of eukaryotes, shortly after the endosymbiosis. The mitochondrial genome of the jakobid lineage apparently experienced the minimum amount of gene transfer from the already substantially reduced mitochondrial genome of the LECA.

Perhaps, the most notable and enigmatic aspect of mitochondrial evolution is the apparent replacement of the four genes for bacterial RNAP subunits that are present in the mitochondrial genomes of jakobids and by inference were contained in the mitochondrial genome of the LECA as well by a single subunit, phage-type RNAP in the rest of the eukaryotes [32,70]. The straightforward interpretation of this replacement is that the jakobids represent the earliest branching clade of the eukaryotes. Under this scenario, the gene encoding the single subunit RNAP was transferred from a prophage in the endosymbiont genome to the nuclear genome at the pre-LECA, stem phase of eukaryote evolution. Subsequently, this gene would have been lost in the jakobid lineage but acquired a mitochondrial import signal and became the mitochondrial RNAP, followed by the loss of the four ancestral RNAP subunit genes, in the lineage leading to the rest of the eukaryotes. The basal position of the excavates or even rooting of the eukaryotic phylogenetic tree within the excavates, with jakobids as the basal branch, is compatible with the latest phylogenetic analysis of a carefully curated set of 37 ancestral eukaryotic proteins of bacterial origin [56]. Our present phylogenetic analysis of the concatenated sequences of the proteins encoded in the mitochondrial genomes (Additional file 1: Figure S5) also separated jakobids from the rest of the eukaryotes (other anomalies in the tree topology notwithstanding). This scenario is compatible with Discicristata (the excavates other than jakobids) being the second most early branching group of eukaryotes (Figure 3c), which would agree with the observed conservation of intron positions in the ATP3 gene that is contained in the mitochondrial genome only in jakobids and *Naegleria gruberi*. Under the jakobid topology, the ATP3 gene was transferred to the nucleus twice, namely, at the branch between the excavates and the rest of the eukaryotes and at the base of the Euglena-Trypanosome branch. The gene encoding the translation factor EF-Tu also contains 2 intron positions that are conserved in more than one eukaryotic supergroup where nuclear copies of the gene have been identified. In this case, however, the evolutionary scenario is complicated by the fact that EF-Tu is encoded in the mitochondrial genome not only in jakobids but also in *V. vermiformis*. The only conceivable evolutionary scenario for this gene includes early transfer of a copy of the EF-Tu gene to the nucleus followed by extended co-existence of the nuclear and mitochondrial copies, with multiple parallel losses of the latter.

An ongoing process of functional gene transfer from the mitochondrial genome to the nuclear genome was observed in angiosperms [42,77,78]. The high frequency of paralogous mitochondrial genes that are encoded both in the nuclear and in the mitochondrial genomes of land plants (Figure 1) is compatible with these observations. Ongoing functional gene transfer from the mitochondrial genome to the nuclear genome is consistent with the observation that in the land plants the intron densities of putative mitochondrial genes are significantly lower than the mean intron density for the corresponding genomes (Figure 4). Apparently, relatively recently transferred mitochondrial genes are still far from having accumulated the saturating intron density. As shown previously, the intron densities of chloroplast-derived genes were slightly albeit significantly lower than those in non-chloroplast-derived genes in land plants [98]. However, the difference observed here for mitochondrial-derived genes is much more dramatic, nearly 1.5-fold in the land plants (Figure 4). Land plant mitochondria are known to have more variable gene content compared to chloroplasts where functional gene transfer from the chloroplast genome to the nuclear genome is a rare event [78]. The present observation suggests that functional gene transfer from the mitochondrial genome to the nuclear genome (and potentially horizontal gene transfer, HGT) is an ongoing process in *Bryophyta* as well. The causes of ongoing functional gene transfer from the mitochondrial genome to the nuclear genome and HGT in land plants but apparently not in other groups of eukaryotes remain unclear.

## Conclusions

Comparative analysis of thousands of mitochondrial genomes across the diversity of eukaryotes validates a previously observed, non-trivial pattern: with very few exceptions, the mitochondrial gene complements of eukaryotes are overlapping subsets of the largest mitochondrial gene set of 66 genes that is found in jakobids. Reconstruction of the evolution of mitochondrial genomes suggests a gene set that was slightly larger than that of jakobids for the last common ancestor of the extant eukaryotes. This superset of mitochondrial genes is much smaller than the gene repertoires of even the simplest known α-proteobacteria and thus represents an intermediate stage in the evolution of eukaryotes that followed extensive loss of genes from the endosymbiont genome at a stage antedating the last common ancestor. The subsequent evolution of mitochondrial genomes in different lineages of eukaryotes consisted primarily in the transfer of ancestral genes, in particular those encoding ribosomal proteins, to the nuclear genomes. Much of this gene transfer occurred in parallel in different lines of evolution. This reconstruction of mitochondrial genome evolution implies that jacobids are the earliest-branching group of eukaryotes that retains some key features of the ancestral endosymbiont such as the multisubunit RNA polymerase. Although not popular previously, this scenario is compatible with the results of some recent phylogenetic studies, and at present appears most plausible.

## Availability of the Supporting Data

Supporting data are available via ftp://ftp.ncbi.nih.gov/pub/koonin/MitoCOGs.

## Additional file

Additional file 1:
**Supplementary information.**
