# Supplementary material for: MitoCOGs: clusters of orthologous genes from mitochondria and implications for the evolution of eukaryotes
Source: BMC Evol Biol. 2014 Nov 25;14:237. doi: 10.1186/s12862-014-0237-5 (PMC4256733; doi:10.1186/s12862-014-0237-5)
Supplement: Additional file 1: — Supplementary information. [file 12862_2014_237_MOESM1_ESM.pdf]

Supplementary Information

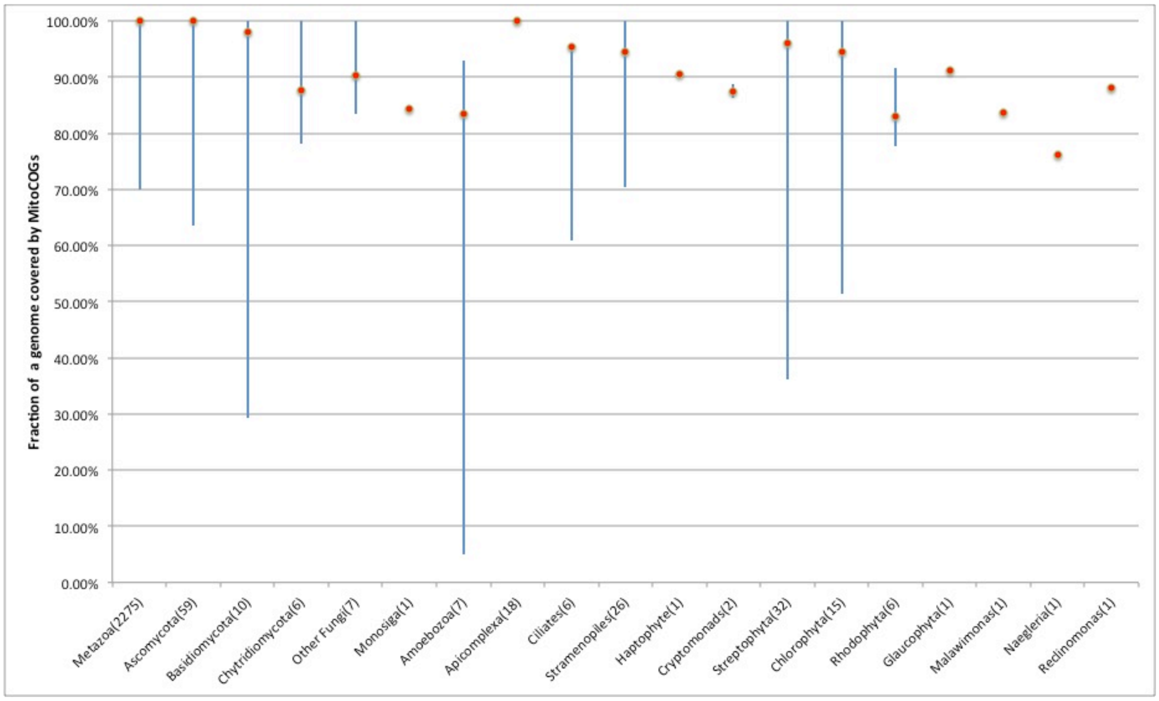

Figure S1. **MitoCOGs coverage of mitochondrial proteomes grouped by taxonomy**. Number of species for each taxonomy group is indicated after the group's name.

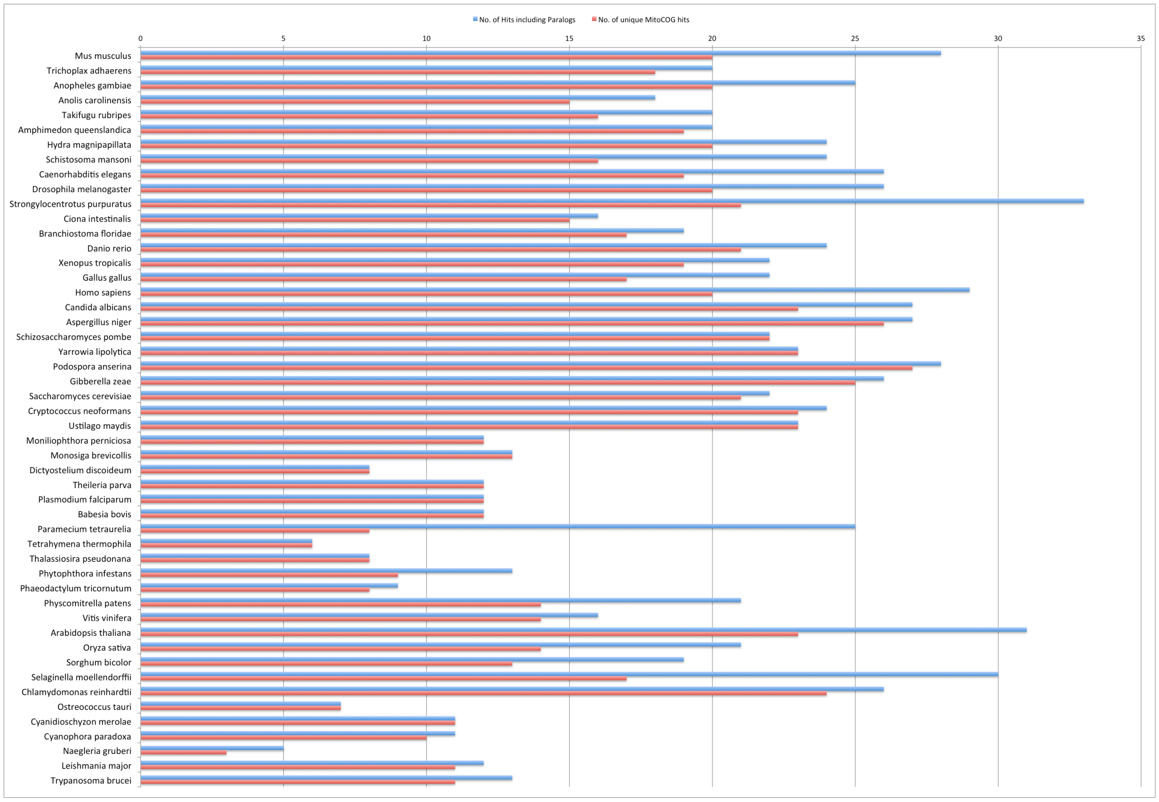

Figure S2. No. of nuclear-encoded sequences (including paralogs and isoforms) in each species (blue) and the unique number of MitoCOGs (red) they map to.

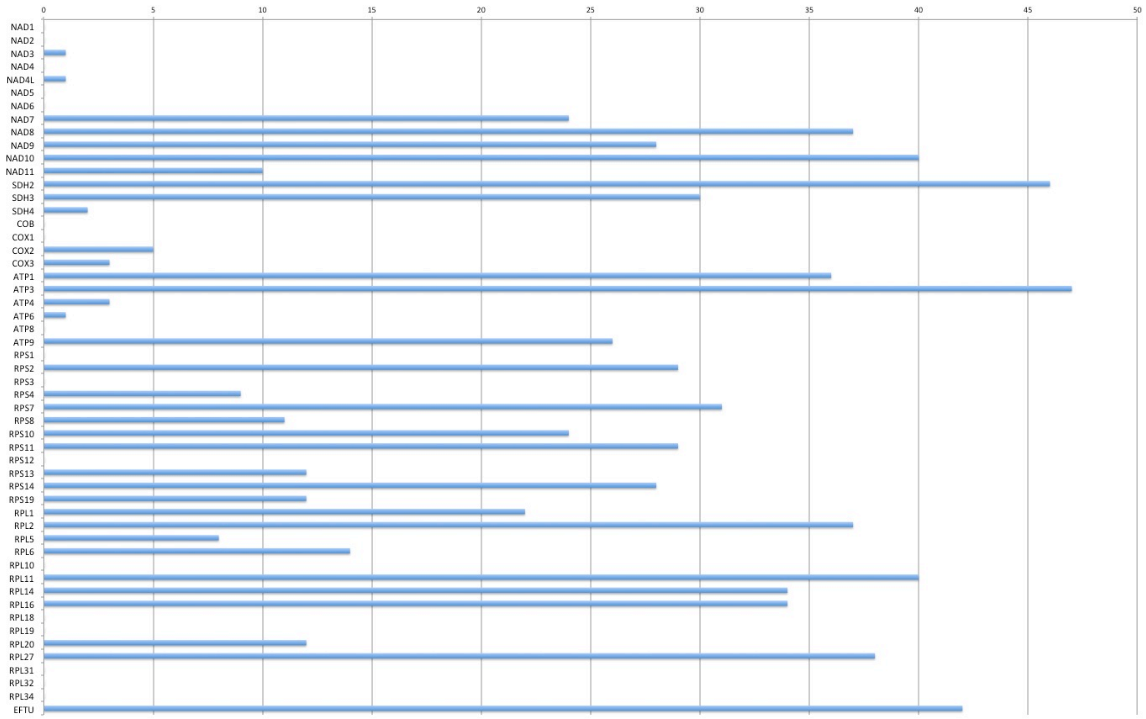

Figure S3. No. of species that have identifiable nuclear-encoded orthologs to MitoCOGs. See Figure 1 for full protein names.

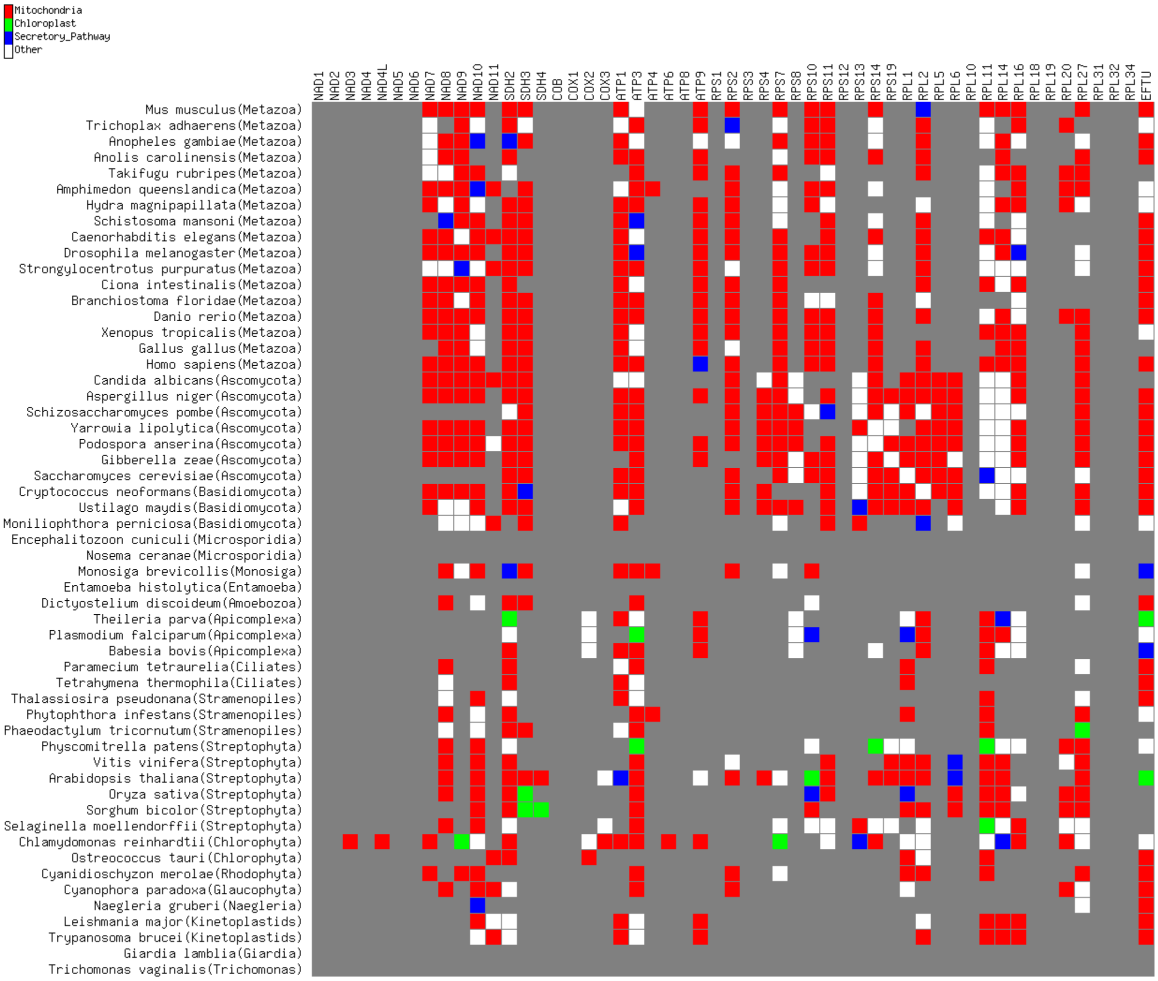

Figure S4 (a). **Subcellular localization prediction for nuclear-encoded orthologs of MitoCOGs using TargetP.** Predicted localizations are mitochondria (red), chloroplast (green), secretory pathway (blue) and any other subcellular compartment (white). Grey color indicates either the protein is encoded in the mitochondrial genome or an orthologous protein is either absent or cannot be identified. See Figure 1 for full protein names.

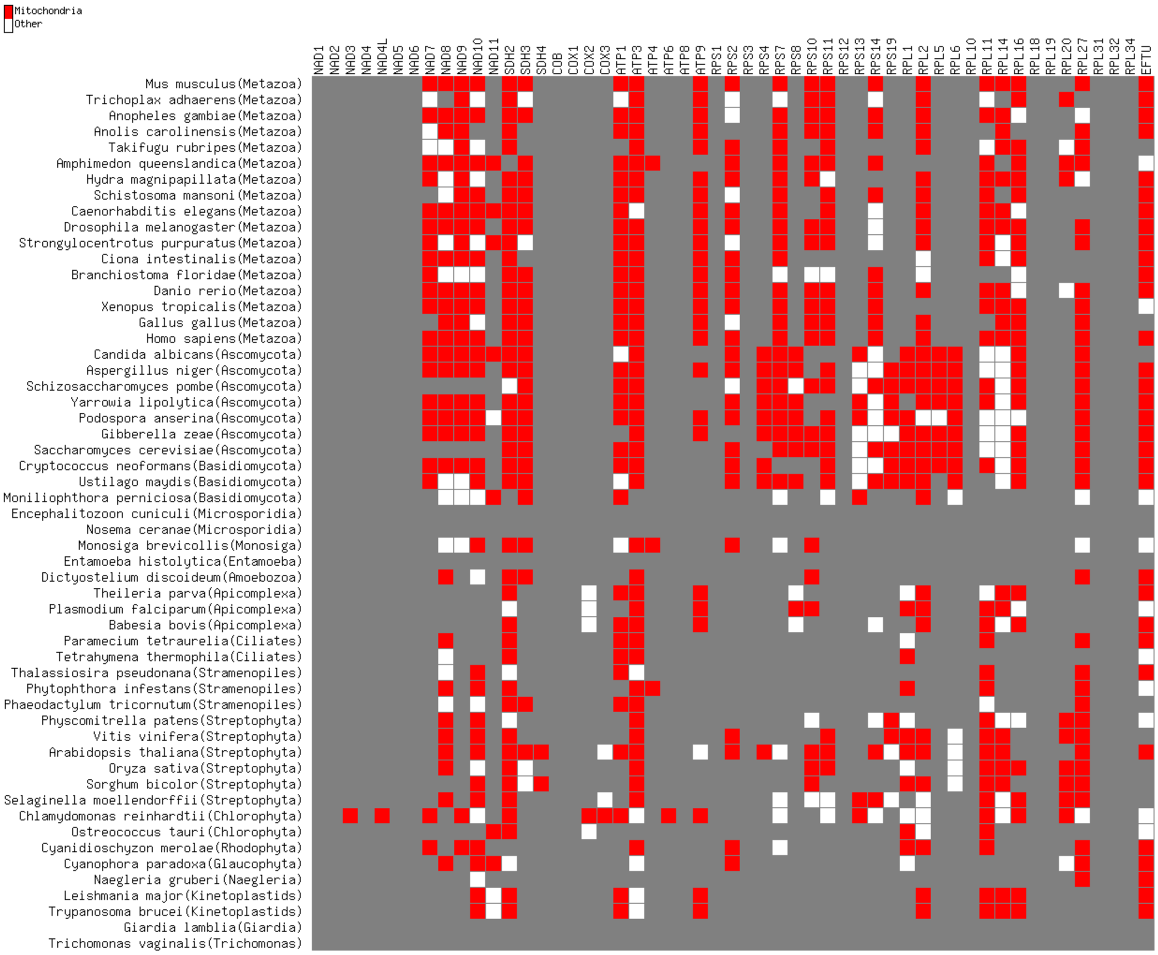

Figure S4 (b). Subcellular localization prediction for nuclear-encoded orthologs of MitoCOGs using MitoProt II. Predicted localizations are mitochondria (red) or any non-mitochondrial compartment (white). Grey color indicates either the protein is encoded in the mitochondrial genome or an orthologous protein is either absent or cannot be identified. See Figure 1 for full protein names.

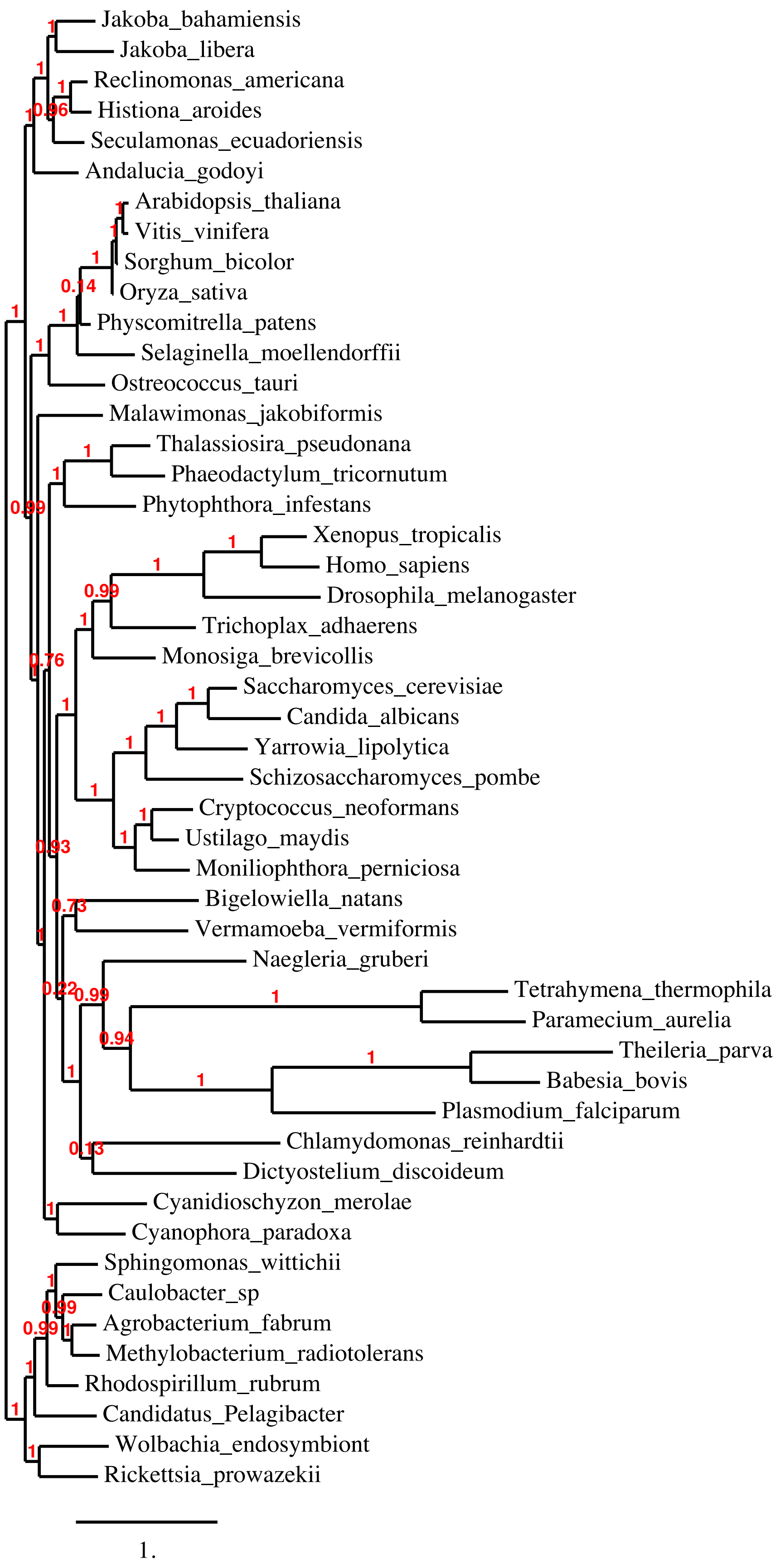
Jakoba_bahamiensis
Jakoba_libera
Reclinomonas_americana
Histiona_aroides
Seculamonas_ecuadoriensis
Andalucia_godoyi
Arabidopsis_thaliana
Vitis_vinifera
Sorghum_bicolor
Oryza_sativa
Physcomitrella_patens
Selaginella_moellendorffii
Ostreococcus_tauri
Malawimonas_jakobiformis
Thalassiosira_pseudonana
Phaeodactylum_tricornutum
Phytophthora_infestans
Xenopus_tropicalis
Homo_sapiens
Drosophila_melanogaster
Trichoplax_adhaerens
Monosiga_brevicollis
Saccharomyces_cerevisiae
Candida_albicans
Yarrowia_lipolytica
Schizosaccharomyces_pombe
Cryptococcus_neoformans
Ustilago_maydis
Moniliophthora_perniciosa
Bigelowiella_natans
Vermamoeba_vermiformis
Naegleria_gruberi
Tetrahymena_thermophila
Paramecium_aurelia
Theileria_parva
Babesia_bovis
Plasmodium_falciparum
Chlamydomonas_reinhardtii
Dictyostelium_discoideum
Cyanidioschyzon_merolae
Cyanophora_paradoxa
Sphingomonas_wittichii
Caulobacter_sp
Agrobacterium_fabrum
Methylobacterium_radiotolerans
Rhodospirillum_rubrum
Candidatus_Pelagibacter
Wolbachia_endosymbiont
Rickettsia_prowazekii
1.

Figure S5. PhyML Maximum likelihood tree generated using concatenated dataset of only MitoCOGs.

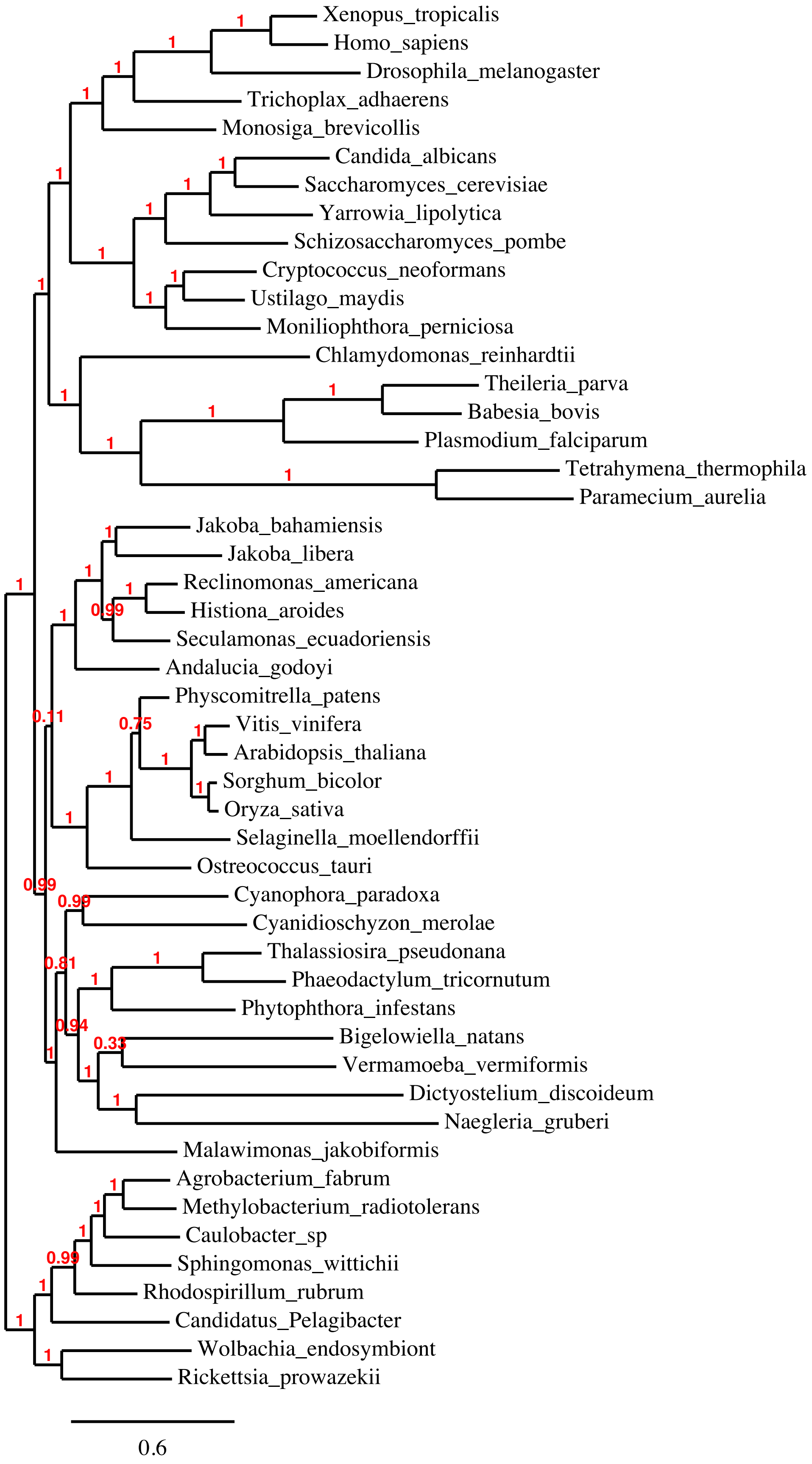
Xenopus_tropicalis
Homo_sapiens
Drosophila_melanogaster
Trichoplax_adhaerens
Monosiga_brevicollis
Candida_albicans
Saccharomyces_cerevisiae
Yarrowia_lipolytica
Schizosaccharomyces_pombe
Cryptococcus_neoformans
Ustilago_maydis
Moniliophthora_perniciosa
Chlamydomonas_reinhardtii
Theileria_parva
Babesia_bovis
Plasmodium_falciparum
Tetrahymena_thermophila
Paramecium_aurelia
Jakoba_bahamiensis
Jakoba_libera
Reclinomonas_americana
Histiona_aroides
Seculamonas_ecuadoriensis
Andalucia_godoyi
Physcomitrella_patens
Vitis_vinifera
Arabidopsis_thaliana
Sorghum_bicolor
Oryza_sativa
Selaginella_moellendorffii
Ostreococcus_tauri
Cyanophora_paradoxa
Cyanidioschyzon_merolae
Thalassiosira_pseudonana
Phaeodactylum_tricornutum
Phytophthora_infestans
Bigelowiella_natans
Vermamoeba_vermiformis
Dictyostelium_discoideum
Naegleria_gruberi
Malawimonas_jakobiformis
Agrobacterium_fabrum
Methylobacterium_radiotolerans
Caulobacter_sp
Sphingomonas_wittichii
Rhodospirillum_rubrum
Candidatus_Pelagibacter
Wolbachia_endosymbiont
Rickettsia_prowazekii
0.6

Figure S6. Maximum likelihood tree generated using concatenated dataset of MitoCOGs and their nuclear-encoded orthologs generated by PhyML

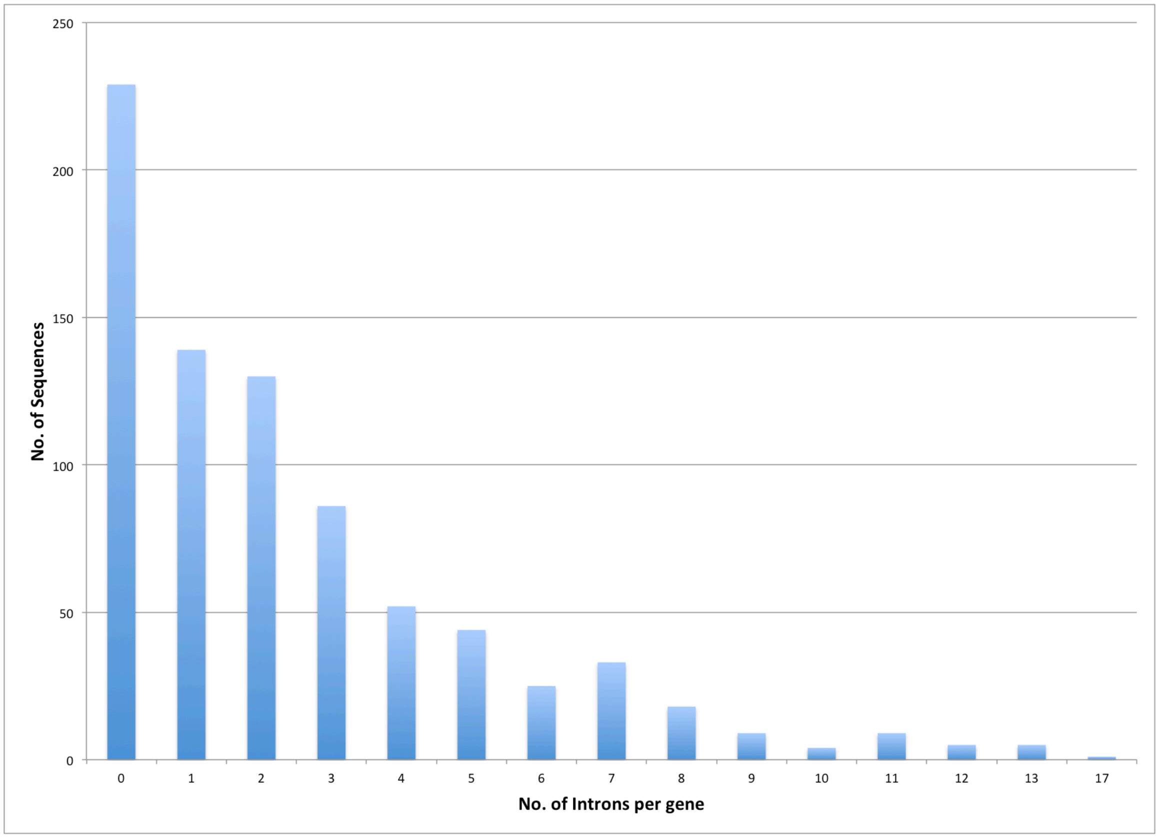

Figure S7. Distribution of intron count per gene for the nuclear-encoded orthologs of MitoCOGs.

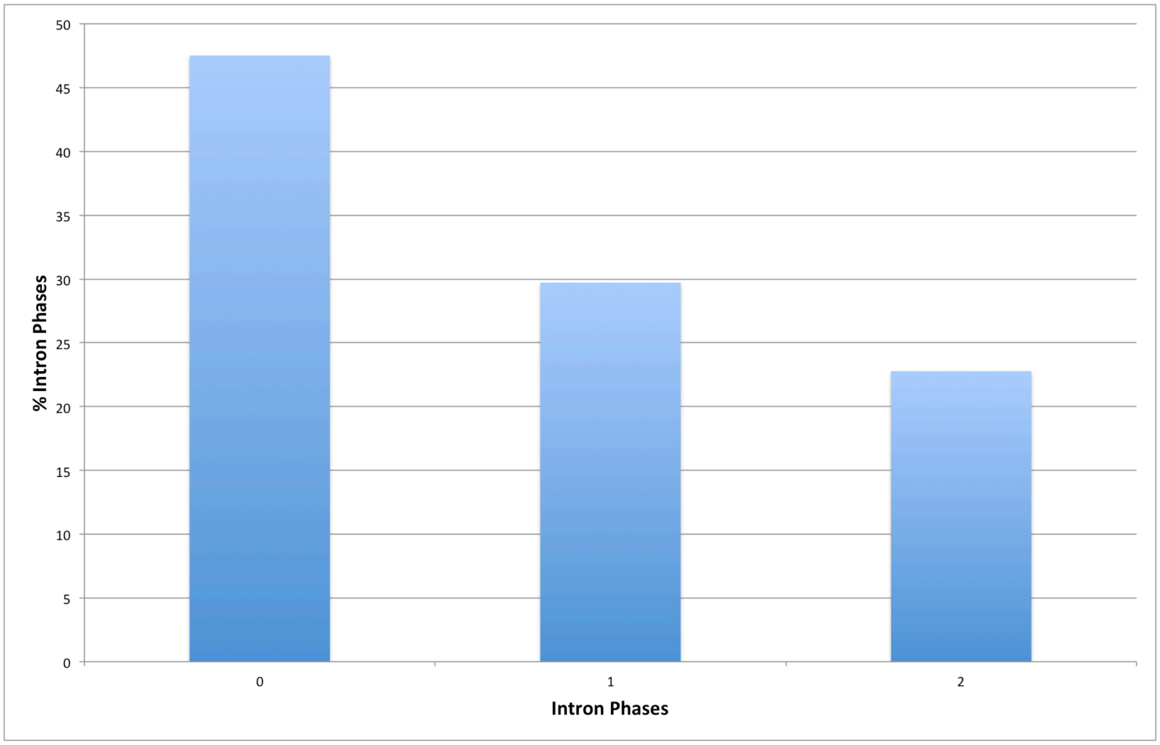

Figure S8. Distribution of intron phases for nuclear-encoded mitochondrial genes

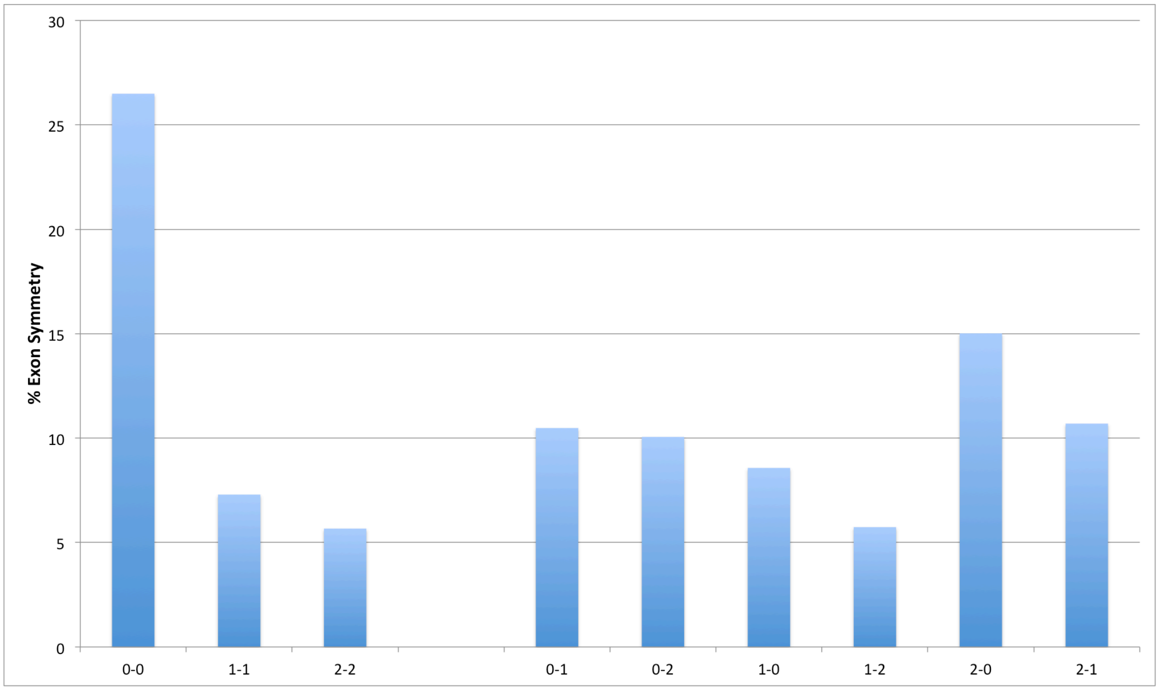

Figure S9. Distribution of exon symmetry for nuclear-encoded mitochondrial genes

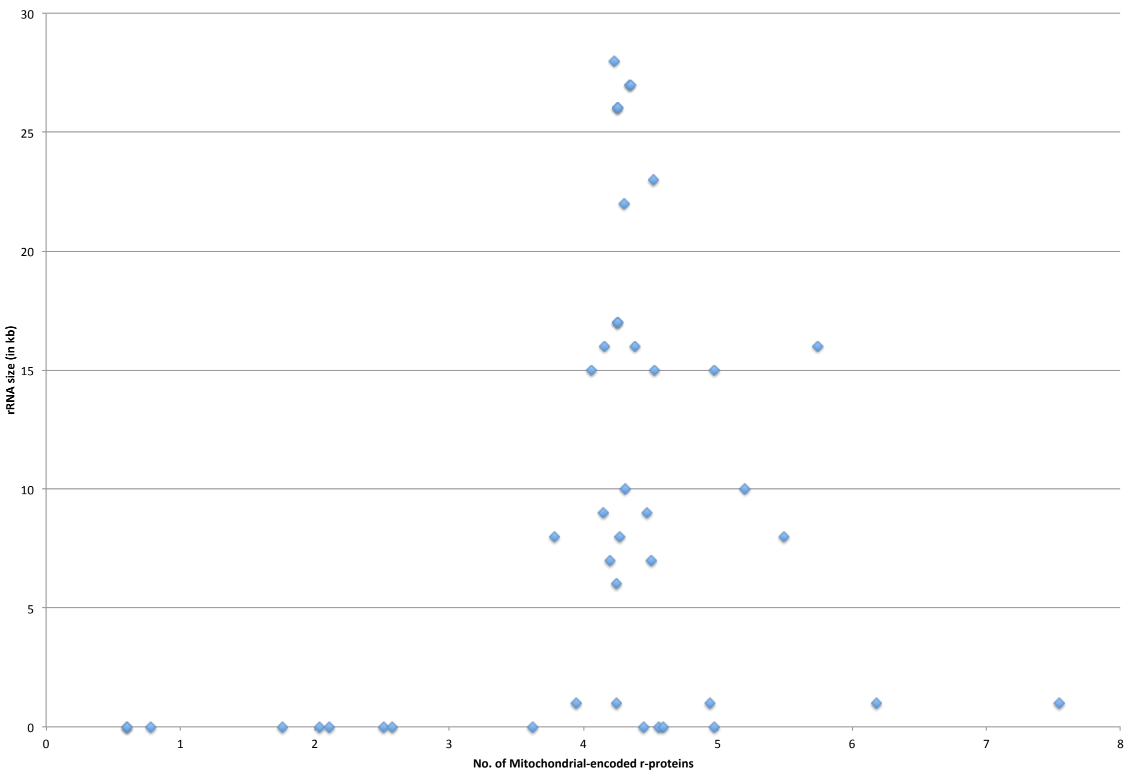

Figure S10. Scatter plot of mitochondrial-encoded ribosomal proteome size and mitochondrial ribosomal RNA size in kb.

Table S1. The 2,486 complete mitochondrial genomes, grouped by taxonomy, used to generate MitoCOGs

Taxonomy group	No. of genomes
**Unikonts**	
Amoebozoa	7
Choanoflagellate	1
Fungi	82
Metazoa	2,275
**Chromalveolates**	
Apicomplexa	18
Ciliates	6
Cryptomonads	2
Haptophyte	1
Stramenopiles	27
**Plantae**	
Chlorophyta	15
Glaucophyta	2
Rhodophyta	6
Streptophyta	33
**Excavates**	
Heterolobosea	1
Jakobids	6
Kinetoplastids*	2
Malawimonads	1
**Rhizaria**	
Chlorarachniophyta	1

*Incomplete genomes

Table S2. Species with poor MitoCOGs coverage

Species	Taxonomic group	Mitochondrial proteome size	No. of proteins in MitoCOGs	Coverage (%)
Physarum polycephalum	Amoebozoa	20	1	5.0
Moniliophthora perniciosa	Basidiomycota	89	26	29.2
Zea mays subsp. mays	Streptophyta	163	59	36.1
Nicotiana tabacum	Streptophyta	156	60	38.4
Arabidopsis thaliana	Streptophyta	117	47	40.1
Nothoceros aenigmaticus	Streptophyta	48	21	43.7
Beta vulgaris subsp. vulgaris	Streptophyta	140	63	45.0
Beta vulgaris subsp. maritima	Streptophyta	150	74	49.3

Table S3. Functional categories represented by MitoCOGs

**Electron transport and ATP synthesis**		
Complex I	NAD 1-11, 4L	12
Complex II	SDH 2-4	3
Complex III	COB	1
Complex IV	COX 1-3	3
Complex V	ATP 1, 3-4, 6, 8-9	6
**Protein translation**		
Small ribosomal proteins	RPS 1-4, 7-8, 10-14, 16, 19, VAR1	15*
Large ribosomal proteins	RPL 1-2, 5-6, 10-11, 14, 16, 18, 19, 20, 27, 31, 32, 34, 35, 36	17
Elongation factor	EFTu	1
**Protein maturation / import**		
Cytochrome c biogenesis	ccmA, ccmB, ccmC, ccmFC, ccmFN	6*
Sec type	SecY	1
Sec-independent	TatA, TatC	2
Cytochrome c oxidase assembly protein	COX11, 15	2
**Transcription**		
RNA polymerase	RpoA,B,C	3
Sigma factor	RpoD	1
**Proteins encoded in mobile elements**		
DNA polymerase		1
RNA polymerase		2
Reverse transcriptase		4
Endonuclease		8
Maturase		2
**Other**		
DNA repair protein	MutS	1
**Function unknown**		49

* RPS3 and ccmB are split in to 2 COGs.

Table S4. List of 55 complete nuclear genomes

Amphimedon queenslandica
Anolis carolinensis
Anopheles gambiae
Arabidopsis thaliana
Aspergillus niger
Babesia bovis
Branchiostoma floridae
Caenorhabditis elegans
Candida albicans
Chlamydomonas reinhardtii
Ciona intestinalis
Cryptococcus neoformans JEC21
Cyanidioschyzon merolae
Cyanophora paradoxa
Danio rerio
Dictyostelium discoideum
Drosophila melanogaster
Encephalitozoon cuniculi
Entamoeba histolytica
Fugu rubripes
Gallus gallus
Giardia lamblia
Gibberella zeae
Homo sapiens
Hydra magnipapillata
Leishmania major
Moniliophthora perniciosa
Monosiga brevicollis
Mus musculus
Naegleria gruberi
Nosema ceranae
Oryza sativa japonica
Ostreococcus tauri
Paramecium tetraurelia
Phaeodactylum tricornutum
Physcomitrella patens
Phytophthora infestans
Plasmodium falciparum 3D7
Podospora anserina
Saccharomyces cerevisiae
Schistosoma mansoni
Schizosaccharomyces pombe
Selaginella moellendorffii
Sorghum bicolor

Strongylocentrotus purpuratus
Tetrahymena thermophila
Thalassiosira pseudonana
Theileria parva
Trichomonas vaginalis
Trichoplax adhaerens
Trypanosoma brucei
Ustilago maydis
Vitis vinifera
Xenopus
Yarrowa lipolytica

Table S5. List of 10 alphaproteobacterial genomes

Agrobacterium tumefaciens C58
Candidatus pelagibacter ubique HTCC1062
Caulobacter K31
Magnetococcus MC 1
Methylobacterium radiotolerans JCM 2831
Rhodobacter sphaeroides ATCC 17025
Rhodospirillum rubrum ATCC 11170
Rickettisa prowazekii BuV67 CWPP
Sphingomonas wittichii RW1
Wolbachia endosymbiont of Drosophila melanogaster

Table S6. List of 5 cyanobacterial genomes

Anabaena variabilis ATCC 29413
Cyanothece PCC 7425
Gloeobacter violaceus PCC 7421
Prochlorococcus marinus MIT 9313
Synechococcus PCC 7002

Table S7. List of 10 archaeal genomes

Aciduliprofundum boonei T469
Aeropyrum pernix K1
Ferroglobus placidus DSM 10642
Halobacterium salinarum R1
Methanocorpusculum labreanum Z
Methanosarcina mazei Go1
Methanothermobacter marburgensis Marburg
Nanoarchaeum equitans Kin4 M
Pyrococcus furiosus DSM 3638
Thermofilum pendens Hrk 5

Table S8. Estimated average propensity of loss for individual mitochondrial encoded proteins and multisubunit complexes from the Count analysis

	Unikont-Bikont Split Topology		Excavates at root Topology		Jakobids at root Topology	
**Protein Name**	**Lost in # lineages**	**Mean of each complex**	**Lost in # lineages**	**Mean of each complex**	**Lost in # lineages**	**Mean of each complex**
ATP1	8.5	5.5	8.5	5.3	8.5	5.4
ATP3	4.1		3.1		4.1	
ATP4	5.2		5.2		5.1	
ATP6	2.4		2.5		2.4	
ATP8	8.5		8.5		8.4	
ATP9	4		4		4	
COB	0	0	0	0	0	0
COX1	0	2.6	0	2.2	0	2
COX2	2		2		2	
COX3	2		2		2	
COX11	4.1		3.1		4.1	
COX15	5.1		4.1		2.1	
NAD1	4	5.1	4	5	4	5.1
NAD2	4		4		4	
NAD3	5		5		5	
NAD4	3		3		3	
NAD4L	5		5		5	
NAD5	3		3		4	
NAD6	5		5		5	
NAD7	6		6		6.1	
NAD8	4		3.1		4.1	
NAD9	5		5		5.1	
NAD10	10.1		10.1		9.3	
NAD11	7.2		7.3		7.1	
RPL1	5.1	6.0	4.1	5.6	2.1	5.1
RPL2	9		9		9	
RPL5	9.5		9.5		9.4	
RPL6	8.1		7.8		7.7	
RPL10	10.2		10.2		9.3	
RPL11	3.1		3.2		3.1	
RPL14	8		8		8	
RPL16	7		7		7	
RPL18	4.1		3.1		4.1	
RPL19	5.1		5.2		5.1	

RPL20	8.2		8.2		8.1	
RPL27	4.1		3.1		1.1	
RPL31	3.1		2.1		3.1	
RPL32	4.1		3.1		1.1	
RPL34	5.1		4.1		2.1	
RPL35	5.1		4.1		2.1	
RPL36	4.1		3.1		4.1	
RPS1	9.1		9.2		9.1	
RPS2	10.8		10.9		10.8	
RPS3	10.5		10.5		10.4	
RPS4	9.5		9.5		9.5	
RPS7	7.5		7.5		7.5	
RPS8	9.5		9.5		9.4	
RPS10	12.5	9.0	12.6	9.1	12.5	9.2
RPS11	8.5		8.6		8.5	
RPS12	6		6		6	
RPS13	9		9		9	
RPS14	10		10		10	
RPS16	3.6		4.2		5.3	
RPS19	11		11		11	
SDH2	6.4		6.4		6.4	
SDH3	10.2	8.3	10.2	8.3	9.3	7.7
SDH4	8.2		8.2		7.3	

Table S9. Taxonomic distribution of lineage-specific MitoCOGs of unknown function

Taxonomic Group	No. of MitoCOGs
Ciliates	19
Streptophytes	18
Stramenopiles	7
Amoebozoa	2
Fungi	1

Table S10. MitoCOGs of unknown function

MitoCOG ID	Taxonomic group	No. of members	Median protein length	Representative Protein GIs	Annotation
MitoCOG0022	Ciliates	6	100	11466253<br>8928580<br>15027630	unknown
MitoCOG0023	Ciliates	5	446	11466254<br>15027631<br>114329813	unknown
MitoCOG0024	Ciliates	5	405	11466255<br>15027632<br>114329814	unknown
MitoCOG0028	Ciliates	4	159	11466260<br>15027637<br>114329819	unknown
MitoCOG0032	Ciliates	6	178	11466266<br>8928606<br>15027643	putative ribosomal protein L6
MitoCOG0033	Ciliates	5	330	11466267<br>15027644<br>114329826	unknown
MitoCOG0034	Ciliates	5	188	11466268<br>15027645<br>114329827	unknown
MitoCOG0035	Ciliates	5	59	11466269<br>15027646<br>114329828	NADH dehydrogenase subunit 1; C-terminal fragment
MitoCOG0036	Ciliates	5	276	11466271<br>15027648<br>114329830	unknown
MitoCOG0037	Ciliates	5	360	11466272<br>15027651<br>114329831	putative NADH dehydrogenase subunit 2?
MitoCOG0038	Ciliates	5	152	11466274<br>15027650<br>114329833	unknown
MitoCOG0041	Ciliates	5	238	11466277	unknown

				15027654<br>114329836	
MitoCOG0042	Ciliates	5	117	11466279<br>15027656<br>114329838	unknown
MitoCOG0044	Ciliates	5	1321	11466282<br>15027660<br>114329842	unknown
MitoCOG0045	Ciliates	5	97	11466286<br>15027664<br>114329846	unknown
MitoCOG0046	Ciliates	5	450	11466287<br>15027665<br>114329847	unknown
MitoCOG0047	Ciliates	6	594	11466288<br>8928587<br>15027666	unknown
MitoCOG0049	Ciliates	5	89	11466294<br>15027674<br>114329854	unknown
MitoCOG0050	Ciliates	5	159	11466296<br>15027673<br>114329856	unknown
MitoCOG0062	Amoebozoa	3	425	7524995<br>60117122<br>87043008	putative ribosomal protein S3; N-terminal fragment
MitoCOG0065	Amoebozoa	3	760	7525005<br>60117132<br>87043034	unknown[1]
MitoCOG0077	Stramenopiles	5	142	9695384<br>50261302<br>145932441	unknown
MitoCOG0078	Stramenopiles	5	225	9695405<br>145932458<br>145932368	unknown
MitoCOG0079	Stramenopiles	5	100	9695412<br>145932465<br>145932375	putative ribosomal protein S4; C-terminus fragment; upstream neighbor protein

					annotated as RPS4 (truncated at C-terminus)
MitoCOG0081	Stramenopiles	11	130	15150729 21450001 114977475	putative ribosomal protein
MitoCOG0083	Stramenopiles	10	378	21450009 84508572 84508612	unknown
MitoCOG0084	Stramenopiles	4	66	145932451 145932361 299830456	unknown
MitoCOG0085	Stramenopiles	5	41	268053536 268053575 268053614	unknown
MitoCOG0089	Streptophytes	9	145	13449344 57013963 323435050	unknown
MitoCOG0094	Streptophytes	3	100	9838387 112253877 323435156	retrotransposon protein, putative, Ty1-copia subclass
MitoCOG0098	Streptophytes	3	124	9838494 57013982 323435130	putative fumarase?
MitoCOG0099	Streptophytes	3	103	57013950 94502680 224365632	Pectinesterase domain containing protein
MitoCOG0100	Streptophytes	6	173	81176529 89280743 94502599	unknown
MitoCOG0101	Streptophytes	3	490	89280744 194033217 289065044	retrotransposon protein, putative, Ty1-copia subclass
MitoCOG0102	Streptophytes	3	181	89280735 194033220 289065046	retrotransposon protein, putative, Ty1-copia subclass

MitoCOG0103	Streptophytes	3	183	89280727 194033223 289065049	similarity to NAD1 at C-terminus
MitoCOG0104	Streptophytes	3	152	89280704 194033214 289065042	putative hydrolase protein
MitoCOG0105	Streptophytes	5	288	89280741 94502593 112253890	unknown
MitoCOG0106	Streptophytes	3	194	89280708 194033227 289065029	unknown
MitoCOG0107	Streptophytes	3	187	89280705 194033237 289065028	unknown
MitoCOG0108	Streptophytes	3	153	89280738 194033239 289065035	unknown
MitoCOG0109	Streptophytes	3	176	89280707 194033242 289065038	putative retrotransposon protein
MitoCOG0110	Streptophytes	3	241	89280719 194033247 289065064	putative 1,3-beta-D-glucan synthase; partial
MitoCOG0111	Streptophytes	3	165	89280737 194033253 289065059	contains pseudo-trnC
MitoCOG0112	Streptophytes	3	284	89280721 194033254 289065058	conserved hypothetical protein; ELF protein
MitoCOG0113	Streptophytes	3	160	89280713 194033263 289065054	similarity to NADH dehydrogenase subunit 4 at N-terminus
MitoCOG0115	Ascomycota	18	432	12408583 18640452 27544883	putative ribosomal protein

Subcellular localization prediction for nuclear-encoded orthologs of MitoCOGs
Mitochondrial proteins, similar to proteins of other organelles, are synthesized in the cytosol and typically are targeted to mitochondria by the N-terminal import signal. Subcellular localization prediction programs rely on the presence of these target peptides to predict the localization. However, a significant fraction of mitochondrial proteins lack typical N-terminal target peptides but instead encompass other, internal targeting signals [1]. In addition, many proteins with N-terminal targeting peptides are dual-targeted to more than one organelle, in particular both to mitochondria and chloroplasts[2]. These factors substantially complicate computational prediction of subcellular localization.

Subcellular localization for the nuclear-encoded mitochondrial proteins was predicted using TargetP and MitoProt II (see Methods). Of the 971 analyzed proteins, TargetP predicted 662 (68%) and MitoProt II predicted 715 (73%) proteins to be targeted to mitochondria (Figures S4a and b), 618 (64%) proteins were predicted to be targeted to mitochondria by both methods, and 769 (79%) proteins were predicted as mitochondrial by at least one method. Although MitoProt II uses hydrophobicity characteristics of the whole protein in addition to the N-terminal sequence used by TargetP, both MitoProt II and TargetP consistently failed to predict mitochondrial targeting for some proteins that are expected to be mitochondria on the basis of orthology (Figures S4a and b). For example, both methods predicted non-mitochondrial localization for the ribosomal proteins S13, L11 and L14 in most fungi.

References

1. Bolender N, Sickmann A, Wagner R, Meisinger C, Pfanner N: **Multiple pathways for sorting mitochondrial precursor proteins**. *EMBO Rep* 2008, **9**(1):42-49.
2. Carrie C, Small I: **A reevaluation of dual-targeting of proteins to mitochondria and chloroplasts**. *Biochim Biophys Acta* 2013, **1833**(2):253-259.
